# metaSPARSim: a 16S rRNA gene sequencing count data simulator

**DOI:** 10.1186/s12859-019-2882-6

**Published:** 2019-11-22

**Authors:** Ilaria Patuzzi, Giacomo Baruzzo, Carmen Losasso, Antonia Ricci, Barbara Di Camillo

**Affiliations:** 1 Department of Information Engineering, University of Padova, via Giovanni Gradenigo, 6, Padova, 35131 Italy; 2 Microbial Ecology Unit, Istituto Zooprofilattico Sperimentale delle Venezie, Viale dell’Università, 10, Legnaro (PD), 35020 Italy; 3 Istituto Zooprofilattico Sperimentale delle Venezie, Viale dell’Università, 10, Legnaro (PD), 35020 Italy

**Keywords:** 16S rRNA, Metagenomic, Simulator, Sparse, Count data, Microbiome, Compositional

## Abstract

**Background:**

In the last few years, 16S rRNA gene sequencing (16S rDNA-seq) has seen a surprisingly rapid increase in election rate as a methodology to perform microbial community studies. Despite the considerable popularity of this technique, an exiguous number of specific tools are currently available for proper 16S rDNA-seq count data preprocessing and simulation. Indeed, the great majority of tools have been developed adapting methodologies previously used for bulk RNA-seq data, with poor assessment of their applicability in the metagenomics field. For such tools and the few ones specifically developed for 16S rDNA-seq data, performance assessment is challenging, mainly due to the complex nature of the data and the lack of realistic simulation models. In fact, to the best of our knowledge, no software thought for data simulation are available to directly obtain synthetic 16S rDNA-seq count tables that properly model heavy sparsity and compositionality typical of these data.

**Results:**

In this paper we present metaSPARSim, a sparse count matrix simulator intended for usage in development of 16S rDNA-seq metagenomic data processing pipelines. metaSPARSim implements a new generative process that models the sequencing process with a Multivariate Hypergeometric distribution in order to realistically simulate 16S rDNA-seq count table, resembling real experimental data compositionality and sparsity. It provides ready-to-use count matrices and comes with the possibility to reproduce different pre-coded scenarios and to estimate simulation parameters from real experimental data. The tool is made available at http://sysbiobig.dei.unipd.it/?q=Software#metaSPARSimand https://gitlab.com/sysbiobig/metasparsim.

**Conclusion:**

metaSPARSim is able to generate count matrices resembling real 16S rDNA-seq data. The availability of count data simulators is extremely valuable both for methods developers, for which a ground truth for tools validation is needed, and for users who want to assess state of the art analysis tools for choosing the most accurate one. Thus, we believe that metaSPARSim is a valuable tool for researchers involved in developing, testing and using robust and reliable data analysis methods in the context of 16S rRNA gene sequencing.

**Electronic supplementary material:**

The online version of this article (10.1186/s12859-019-2882-6) contains supplementary material, which is available to authorized users.

## Background

Next generation sequencing (NGS) has now become the most widely used approach to perform microbial community studies. In particular, the discovery of the 16S ribosomial RNA universal marker gene and the ever-decreasing experimental costs of sequencing, made it the most adopted method for taxonomic studies [1–3]. Among all NGS methodologies, targeted amplicon sequencing of the 16S ribosomial RNA (16S rRNA) gene, referred to as “16S rDNA-seq” from here on, is currently one of the most used strategies for the identification and quantification of microbial population residing in a specific ecological niche. The 16S rRNA gene was chosen as a target because of its ubiquitous presence in prokaryotes and its natural structure that is made from both highly conserved and highly variable regions. The conserved regions are used as amplification targets for polymerase chain reaction (PCR) universal primers to select one or more hypervariable regions of the 16S rRNA gene [4–6]. The variable regions are species-specific regions that demonstrate considerable sequence diversity among different bacteria and consequently allow for taxonomic classification and phylogenetic analysis. After targeted amplification, the obtained fragments undergo the sequencing process from which thousands of nucleotidic sequences, called “reads”, are obtained. Then, reads are usually clustered according to their sequence similarity into the so-called “operational taxonomic units” (OTUs) or into Amplicon Sequence Variants (ASVs). The number of reads corresponding to each OTU/ASV is used as a proxy of the abundance of that feature in the original sample and results are summarized in a matrix known as “OTU table”. In this table, OTUs are organized by row and samples by column and each matrix entry is filled with the number of reads belonging to each OTU within each sample. Throughout the manuscript, we will refer to these numbers as “counts” and we will use the terms “taxon”, “OTU”, “ASV”, “species” and “feature” interchangeably to identify the rows of these matrices. Additionally, many experimental conditions (also called “groups” in this work) are usually present within the same experiment; for example, samples from “treated” vs “control” individuals or samples from different body sites may be present. Samples belonging to the same group are referred to as biological replicates.

The analysis of 16S count data is usually performed using either methods developed for RNA sequencing or methods, when available, specifically developed for 16S count data. Benchmarking these different methods against common gold standards would be a good practice to identify the best analysis pipelines and to improve the quality of data analysis methods.

However, 16S rDNA-seq data characteristics, such as strong sparsity and variability levels, make their simulation challenging, also due to the difficulty of choosing the most appropriate generating model. In the following sections, we first report a brief excursus of the models that have been proposed in the literature for modelling count data and then, we summarize 16S rDNA-seq data simulation state-of-the-art. Finally, we introduce metaSPARSim, a stand alone tool specifically proposed for 16S count data simulation.

### Modelling sequencing count data

In the past years, several models were proposed to describe the nature of sequencing count data. A classical approach for modelling count data is by using a Poisson random variable, *Y*_*ij*_, whose parameter *λ*_*ik*_ represents the mean expected count value for feature *i* in experimental group *k* to which sample *j* belongs. The above model has a well known main characteristic, that is the mean equals the variance. This modellization is based on the fact that a DNA sample can be seen as a collection of fragments taken from the species present within it and then DNA sequencing can be compared to a random sampling of the species, with the aim of estimating the relative abundance of each species in the niche. If we think each DNA fragment like having the same chance of being selected for sequencing and the fragments being selected independently, then the number of counts for a given feature in repeated measurements could be described with a Poisson variation law. The consistency of this hypothesis has been examined in Marioni et al. [7], in which the same initial collection of RNA distributed across multiple lanes of Genome Analyzer (Illumina) sequencer was used. In this work, the Poisson model turned out to be a good description of technical replicates for most of the features. When biological replicates are also considered, the count variance is observed to be higher then the mean (the so-called over-dispersion phenomenon) due to the fact that the number of fragments for the same species among different samples is affected by biological variability. Therefore, to describe the biological plus technical variability, another well established approach based on the Negative Binomial (NB) distribution has been adopted in sequencing count data modellization [8, 9]. The NB arises as a compound probability distribution where the distribution of the Poisson rate *λ*_*ik*_ is described by a gamma distribution, which is why the NB is also called Poisson-gamma mixture distribution. Due to the extra-variation introduced by the gamma component, the resulting distribution then acts like an over-dispersed Poisson model. Indeed, according to this model the total variance of *Y*_*ij*_ is:

1 $$ Var(Y_{ij})=\lambda_{ik}(1+\lambda_{ik}\phi_{ik}),  $$

where *ϕ*_*ik*_ is known as the dispersion parameter. When *ϕ*_*ik*_ goes to zero, the variance of *Y*_*ij*_ equals its mean, thus obtaining again the Poisson distribution.

Both the above modellizations come from bulk RNA sequencing count data context and their translation in 16S rDNA-seq framework is not straightforward. In fact, 16S rDNA-seq OTU tables share with RNA-seq count data some main characteristics, such that of being non-negative and over-dispersed, but they are typically affected by a high number of zeros. The latter phenomenon is known as “zero-inflation”. As recalled in Xu et al. [10], one way to deal with such a big amount of zeros in count data is to use zero-inflated (ZI) models [11], which are basically mixtures of Poisson (ZIP) or Negative Binomial (ZINB) models with a point mass at zero. Another approach is to use a hurdle model [12], a model formed by two parts, the first being modelled by a binomial distribution used to determine whether a zero or non-zero outcome occurs, and the second being a count data modelling truncated at zero to characterize positive counts. The main difference between the two approaches lies in the fact that ZI models assume that the zero observations have two different origins: “structural” and “random”. The first zero values are real zeros that indicate the absence of the feature in the sample; whereas, random zeros are caused by insufficient sequencing depth when performing sequencing, thus causing rare taxa to be dropped from the sequenced population. On the contrary, hurdle models do not make the distinction between structural and sampling zeros, assuming that all zero data are from one unique structural source. The idea is that positive counts occur once a threshold is crossed, or a hurdle is cleared. If the hurdle is not cleared, then we have a count of 0.

It is noteworthy that the above models consider all the features present in a sample as being independent one from each other, thus ignoring the sum constraint imposed by the fact that sequencing platforms can produce reads only up to their capacity (i.e. the sequencing depth). Indeed, it is now well established that sequencing data only carry relative information [13, 14], i.e. count tables entries are not actually absolute counts, but rather portions of a whole. This characteristic, known in the compositional data analysis framework as *scale invariance* [15], is in contrast with the assumption of OTU independence and, consequently, with the simulation of count tables modelling single taxa separately [16]. To deal with this limitation, Dirichlet-multinomial [17, 18] and logistic normal multinomial models [19] have been proposed for modelling all features simultaneously based on their relative abundances.

### Simulation of 16S rDNA-seq count tables in the literature

Most of the available tools in the literature intended for 16S rDNA-seq data simulation are focused on producing synthetic reads [20, 21]. While such tools are very useful for read-level tasks (e.g. read quality filtering, sequences clustering, etc.), their main goal is generating realistic nucleotide sequences, more than achieving realistic reads abundances. In addition, simulated reads must be processed to obtain the final OTU table, requiring additional and computational intensive steps.

The availability of simulators for synthetic count data generation is of pivotal importance for all those researchers who deal with benchmarking or testing procedures to assess the performance of available preprocessing and analysis methods. To serve this function, a simulator has to encompass some key characteristics. First, its code has to be available for users in a direct way, such that the simulating procedure is encoded and easily reproducible by anyone. Second, it should be accompanied by a user guide in which the simulation procedure is clearly explained so that misuses of the tool are avoided. Third, input parameters should be provided either directly or indirectly (e.g. by automatic estimation from a real dataset) in order not to leave the user the full charge of making assumptions and hypothesis on them. Lastly, but very important, the simulator should have been tested before the release and a sufficient evaluation of its performance should be available before releasing it to the scientific community. To the best of our knowledge, no simulator with the above mentioned features is now available in the literature for a user-friendly simulation of 16S rDNA-seq count tables. Indeed, the distributions used to model 16S data were originally proposed as modelling frameworks addressing data analysis issues (clustering, classification, variable selection, etc.) [17–19] and not with the aim of giving researchers a tool for synthetic data generation. Therefore, no comprehensive assessment of goodness of simulated data via comparison with real experimental data was included in the original works. Additionally, even though several benchmarking papers are based on these modellizations ([22–24]), each of these studies includes its own synthetic data simulation, and no simulating tool with an appropriate guide for data simulation has been released within these works. For example, in Chen et al. work [25] a novel test for differential distribution analysis of microbiome sequencing data is proposed and the authors use the ZINB model to simulate synthetic datasets for testing their method performance. In Chen et al., only a part of the R scripts used for simulation is freely accessible, mainly for illustrative purpose. Another work, by Kurtz et al. [26], proposes a statistical method for the inference of microbial ecological networks from 16S rDNA-seq data accompanied by computational tools able to generate OTU count data. The simulation procedure is included within the ecological networks inference tool package. However, the related tutorial only shows how to launch a zero-inflated negative binomial simulation with some specified parameters and very little space was given in the paper to assess simulation performance.

### Rationale of our work

Our simulator is proposed as a stand-alone tool intended for simulating synthetic 16S count tables to be used for the assessment of tools for preprocessing and downstream analysis, such as count data normalization, zero-values imputation, differential abundance testing and so on. metaSPARSim is freely accessible and equipped with a user guide where examples and applications to possible benchmarking frameworks are included to facilitate scientists in the usage of its estimation and simulation functionalities.

In this article, the Multivariate hypergeometric (MHG) distribution is exploited to accurately model 16S rDNA-seq count data. The proposed modellization is included in metaSPARSim, a novel 16S rDNA-seq count table simulator implemented as an R package (see below). We model 16S gene sequencing as a sampling process with limited number of extractions and not independent sampling, i.e. under a multivariate framework. Contrarily to the above mentioned multinomial models, our model considers sampling modellization of the sequencing procedure without replacement; this reflects the fact that the probability that a read comes from a precise bacterial agent is dependent on the abundance of that and the other agents within the total population, but it is variable during sequencing, because when a fragment is captured and read, it is no longer available for other binding sites. That is, one member of that bacterial class is no longer available for the future drawings.

In the literature MHG distribution is approximated by the Multinomial in many applications and sampling with or without replacement are converging models when the population size and the sampling size are sufficiently far away one from each other so that different fragment sampling probabilities can be considered as constants. However, the shape of the population class abundance vector distribution greatly influences the allowed magnitude of the gap between population and sampling size for Multinomial-MHG convergence and therefore it does not exist a unique and standard cut-off to numerically guarantee the legitimacy and applicability of this approximation. Indeed, the replacement effect on a uniformly distributed and on a strongly-skewed distribution would be very different. The proposed MHG modellization overcomes the problem of verifying if the conditions for multinomial approximation hold, providing a framework that is theoretically more rigorous and realistic to model real abundance inter-dependency.

To obtain typical overdispersion detected in 16S rRNA data, the parameters defining internal classes subdivision for the MHG distribution are modelled according to a gamma distribution.

## Methods

### The model

metaSPARSim simulation is based on a two-steps gamma-MHG model: first, species abundances varying between biological replicates are modeled using a gamma distribution; second, the technical variability originated by the sequencing process is modelled using a Multivariate Hypergeometric model.

In particular, let *C* be the number of observed features (OTUs) and **Y**_*j*_ the vector of counts *Y*_*ij*_ for feature *i* in sample *j* belonging to experimental group *k*. Then we can write:

2 $$ \begin{aligned} \mathbf{Y}_{j}\sim MHG\left(n_{j},\mathbf{m}_{j}\right), \\ m_{ij}\sim Gamma\left(\frac{1}{\phi_{ik}},\phi_{ik}\cdot\mu_{ik}\right). \end{aligned}  $$

where

- *n*_*j*_ is the library size of sample *j*
- **m**_*j*_=(*m*_1*j*_,*m*_2*j*_,…,*m*_*Cj*_) is the vector indicating the number of fragments that are available in sample *j* for OTU *i* before sequencing
- *μ*_*ik*_ is the “average” abundance level of OTU *i* in group *k*, to which sample *j* belongs
- *ϕ*_*ik*_ is a parameter describing the biological variability in the abundance level of OTU *i* in group *k*, to which sample *j* belongs.

### The tool

metaSPARSim is a tool written in *R* language with core function implementation in *C++* (*C++* code was integrated into the R script using *Rcpp* library). It generates datasets with a number of experimental groups and biological replicates specified by the user in input. For each sample group, the simulation takes as input a vector of species abundances, together with a measure of biological variability. These parameters can be specified by the user, estimated from a real dataset or taken from a set of pre-coded scenarios that are integrated into the simulator. For each OTU, a gamma distribution with specified mean and variance is used to generate species abundances in different replicates. Then, the sequencing step is reproduced by sampling the wanted sequencing depth from each biological replicate accordingly to a MHG distribution, whose internal probabilities are defined by sample-specific proportional abundances.

In the following, metaSPARSim inputs, outputs and available precoded datasets are presented.

#### Inputs

The simulation of 16S rDNA-seq data needs as input a pair of vectors (*μ*_*ik*_ and *ϕ*_*ik*_) modelling abundance and biological variance for each group *k* and OTU *i* (see Eq. 2). In addition, the library size for each simulated sample is needed. To specify the above parameters, the user can choose among three different input modes, as follows. Case 1. **Direct specification** In the first modality, the user can specify his/her own parameters. Once the number of sample groups is fixed, the only compulsory parameters to give the simulator for each group are:

- One vector of species abundances, (*μ*_1*k*_,…,*μ*_*Ck*_)
- One vector of species abundance variability, (*ϕ*_1*k*_,…,*ϕ*_*Ck*_)
- One vector of desired library sizes, $(n_{1}, \dots, n_{n_{k}})$, with *n*_*k*_ the total number of samples in experimental group *k*.

Case 2. **Estimation procedure from real datasets** metaSPARSim also allows for estimation of parameters from real count tables. Obviously, it is not possible to reconstruct real absolute abundances of OTUs from a real dataset. However, a raw estimate to be used as input for the simulation is sufficient, since our purpose here is to obtain from a known input a count table which resembles the characteristics of a real dataset.

Given a real count matrix, metaSPARSim internally estimates species abundances, species abundance variabilities and library size vectors for simulation by the use of built-in functions (see Additional file 1). Additionally, also the hybrid mode is available. For example, one may want to take information about mean values from a real experiment while using personally specified vectors of species abundance variabilities or library sizes. Case 3. **Available presets included in the simulator** The user can simulate his/her own 16S count matrix by taking advantage of the pre-coded scenarios present in the simulator (see Additional file 1: Table S1). In fact, different sets of parameters taken from real datasets or synthetically designed to describe theoretical distributions of interest in microbiome studies are available in our tool.

#### Outputs

metaSPARSim outputs its results as a list composed by two elements:

- A matrix containing simulated count data organized with features (OTUs, ASVs, …) on rows and samples on columns. This has to be intended as the count table coming from a simulated sequencing experiment, i.e. a synthetic analogue of an OTU table coming from a targeted microbiome sequencing run.
- A matrix with features (OTUs, ASVs, …) on rows and samples on columns containing sample relative abundance values $\left (m_{ij}/{\sum \nolimits }_{i} m_{ij}\right)$ after the first simulation step of biological variability using the gamma distribution. Despite not being the main result of the simulation procedure, this intermediate output is a fundamental tool for users who want to perform benchmarking on preprocessing/analysis methods dealing with sequencing bias correction or recognition, because it encloses the information about the simulated microbiomes composition before the sequencing step simulation with MHG sampling. This should be indeed intended as the ground truth values to use as golden standard when comparing data processed with, for example, different normalization, zero-imputation or differential abundance tools.

### Test datasets and evaluation criteria

The goodness of metaSPARSim simulations was assessed using both publicly available datasets, such as Human Microbiome Project (HMP) data [27, 28], and two proprietary datasets monitoring animal gut and raw milk cheese microbial communities. The main characteristics of the real datasets used for comparison are shown in Table 1 (additional details can be found in Additional file 1). These datasets were chosen to obtain a wide range of different scenarios, including experiments based on different sequencing platforms and with different group, sample and replicate numbers and sequencing depths.

**Table 1:** Real datasets characteristics

Characteristic	Animal gut	Raw milk cheese	HMP
*Samples*	110	118	40
*Replicates*	5	2-3	5
*Groups*	22	40	8
*Features*	3541	3109	758
*Sequencing depth (range)*	88692-832309	28536-349754	2798-24095
*Count data sparsity*	78.69%	97.12%	81.37%

To explore metaSPARSim performance, a comparison between real and simulated data was performed. In particular, we compared real and simulated data in terms of:

- **Sparsity:** the percentage of zero counts per row, per column, and in total;
- **Intensity:** the normalized (*scranR* package [29] was used) count value intensity averaged across replicates within the same group;
- **Variability:** the variance and the relative variance of normalized count values, calculated across replicates within the same group.

A Mann-Whitney U test was performed to test for group mean distribution differences between real experimental count tables and simulated count tables for each dataset. Additionally, the related effect size was calculated and a bootstrap procedure (10000 extractions with 5% of total feature number) paired with a Mann-Whitney U test was performed to test for significance in subsamplings, thus overcoming sampling size issue for significance (see Additional file 1 for further details).

## Results

***Sparsity***

**Fig. 1:**
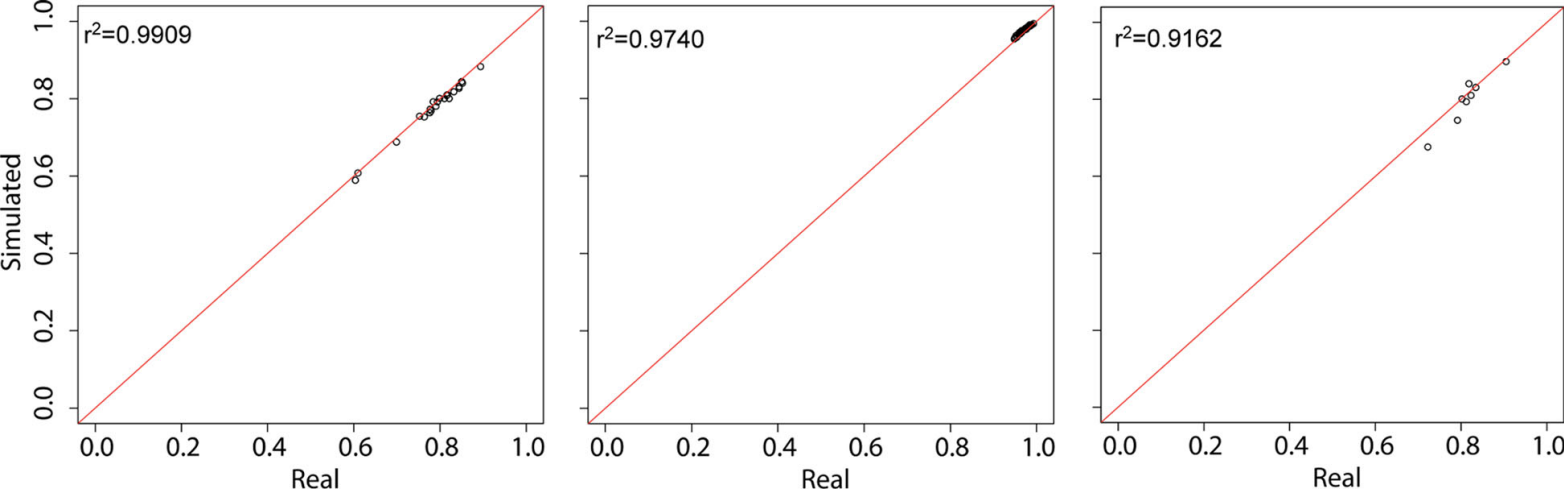
Group-specific sparsity in real and simulated data. Scatter plot of group-specific percentage of zeros in real and simulated datasets. From the left, animal gut, raw milk cheese and HMP data results. *R*^2^ values are also reported

**Fig. 2:**
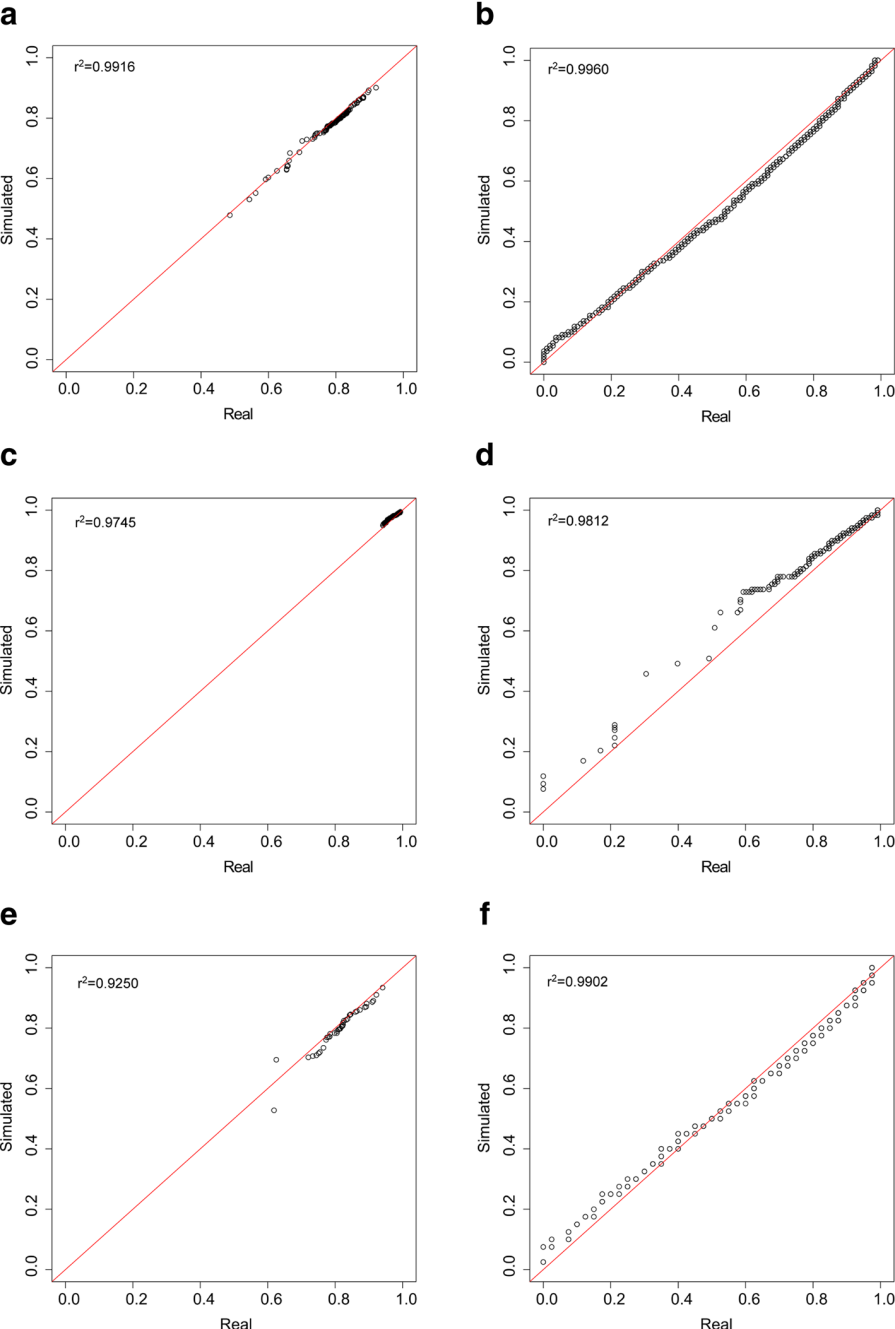
Sample-wise and feature-wise sparsity in real and simulated data. Q −Q plot of percentage of zeros in real and simulated datasets, calculated by sample (**a**, **c**, **e**) and by feature (**b**, **d**, **f**) for animal gut (first row), raw milk cheese (second row) and HMP (third row) data. *R*^2^ values are also reported

The first examined metric was the level of sparsity of the simulated matrix. The overall zero abundances were very well reproduced in all the datasets, the real ones being of 78.7%, 97.1% and 81.4% and the simulated ones of 77.9%, 93.8% and 80% respectively for animal gut, raw milk cheese and HMP data. For all the three datasets, in Fig. 1 the true sparsity percentages calculated on group submatrices is plotted against the simulated ones. As can be seen, all the results confirm that the accuracy in recreating datasets with realistic overall sparsity remains valid when looking at intra-group sparsity. Additionally, zeros-by-row (feature) and zeros-by-column (samples) distributions were calculated to check if the simulator was able to reconstruct not only the true zero abundance but also the true location of zero counts. As showed in Fig. 2, the zero distributions per row and per column are well reproduced in all the datasets.

***Intensity***

**Fig. 3:**
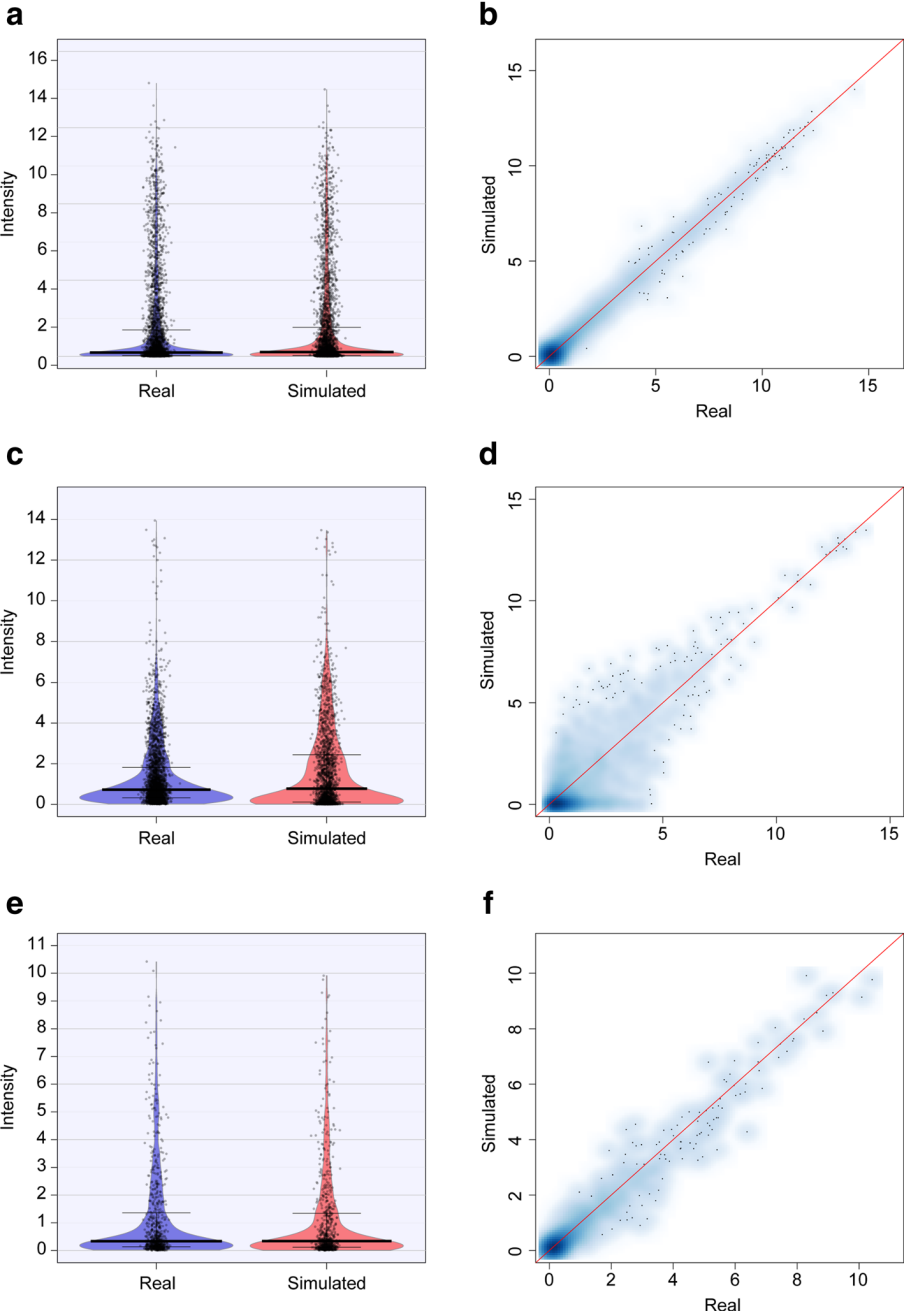
Count intensity in real and simulated data. Comparison of Log2 count mean intensity in real and simulated datasets, represented as RDI plot (**a**, **c**, **e**) and scatter plot (**b**, **d**, **f**) for animal gut (first row), raw milk cheese (second row) and HMP (third row) data, excluding zero mean features

Count values in real and simulated data showed very similar characteristics, as shown by RDI (Raw (data), Description and Inference) plots (Fig. 3a, c, e) and scatter plots (Fig. 3b, d, f). The performances observed for whole count data were maintained when looking at the intensity within each group (Additional file 1: Figures S4–S12), assuring that the overall good behaviour was the result of a good performance in each single group.

Results of the statistical tests (shown in Additional file 1: Tables S2–S4) confirmed real and simulated values came from the same distribution. In fact, although many groups showed significant differences for the global test, the related effect size was always found to be negligible and the percentage of bootstrap extractions in which significance was found was null for all the groups, thus confirming significance was strongly due to the huge sample sizes (number of features).

**Fig. 4:**
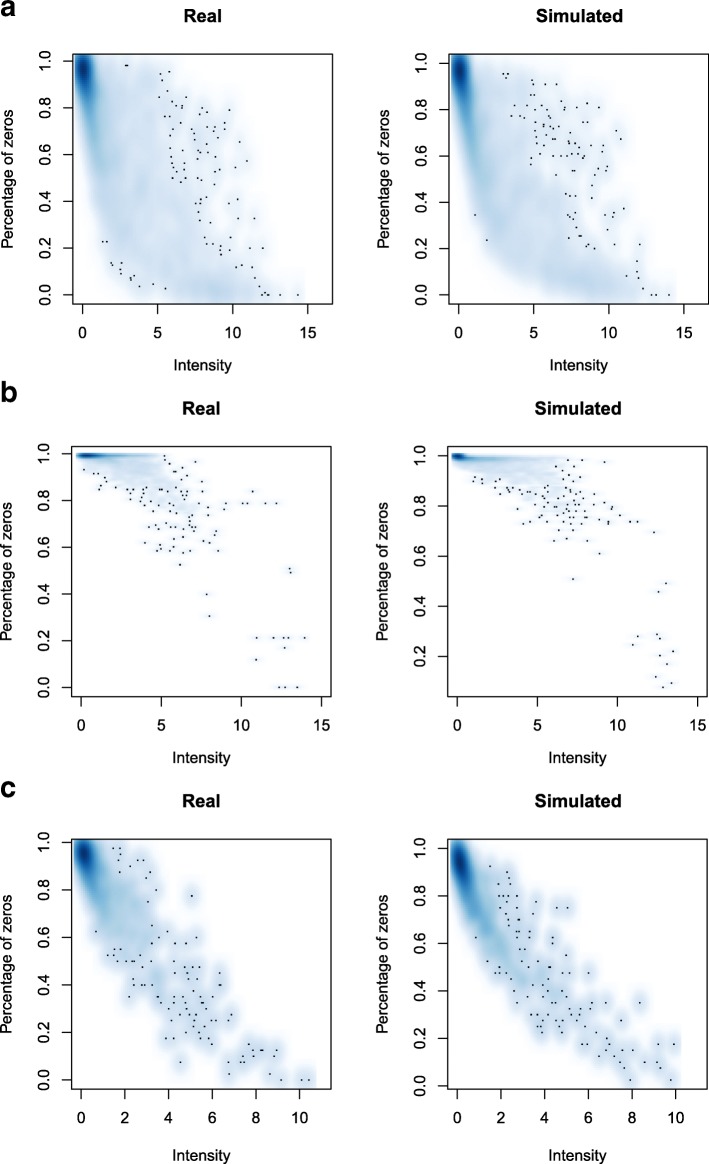
Intensity-sparsity relation in real and simulated data. Scatter plot of the relation between Log2 count mean intensity and sparsity in real and simulated datasets, for animal gut (**a**), raw milk cheese (**b**) and HMP (**c**) data, excluding zero mean features

To further investigate the ability to reproduce real experimental data structure and characteristics, the relation between the first two metrics (intensity and sparsity) was studied. As reported in Fig. 4, the dependency between mean intensity and sparsity was accurately maintained from real to simulated datasets, for all the three investigated cases.

**Variability**

For variability metric, results are shown in terms of both variance and relative variance (RV, or variance-to-mean ratio), i.e.

3 $$ RV=\frac{\sigma^{2}}{\mu}.  $$

As for intensity, metaSPARSim was able to reproduce in a realistic way data variability in HMP and animal gut datasets, while for raw milk cheese data it was able to capture the median values (Fig. 5 and Additional file 1: Figures S14–S25). However, no statistical difference was found between real and simulated data variance, the effect sizes being always negligible and the bootstrap percentage of significant tests always being under the 9% (Additional file 1: Tables S5–S7).

**Fig. 5:**
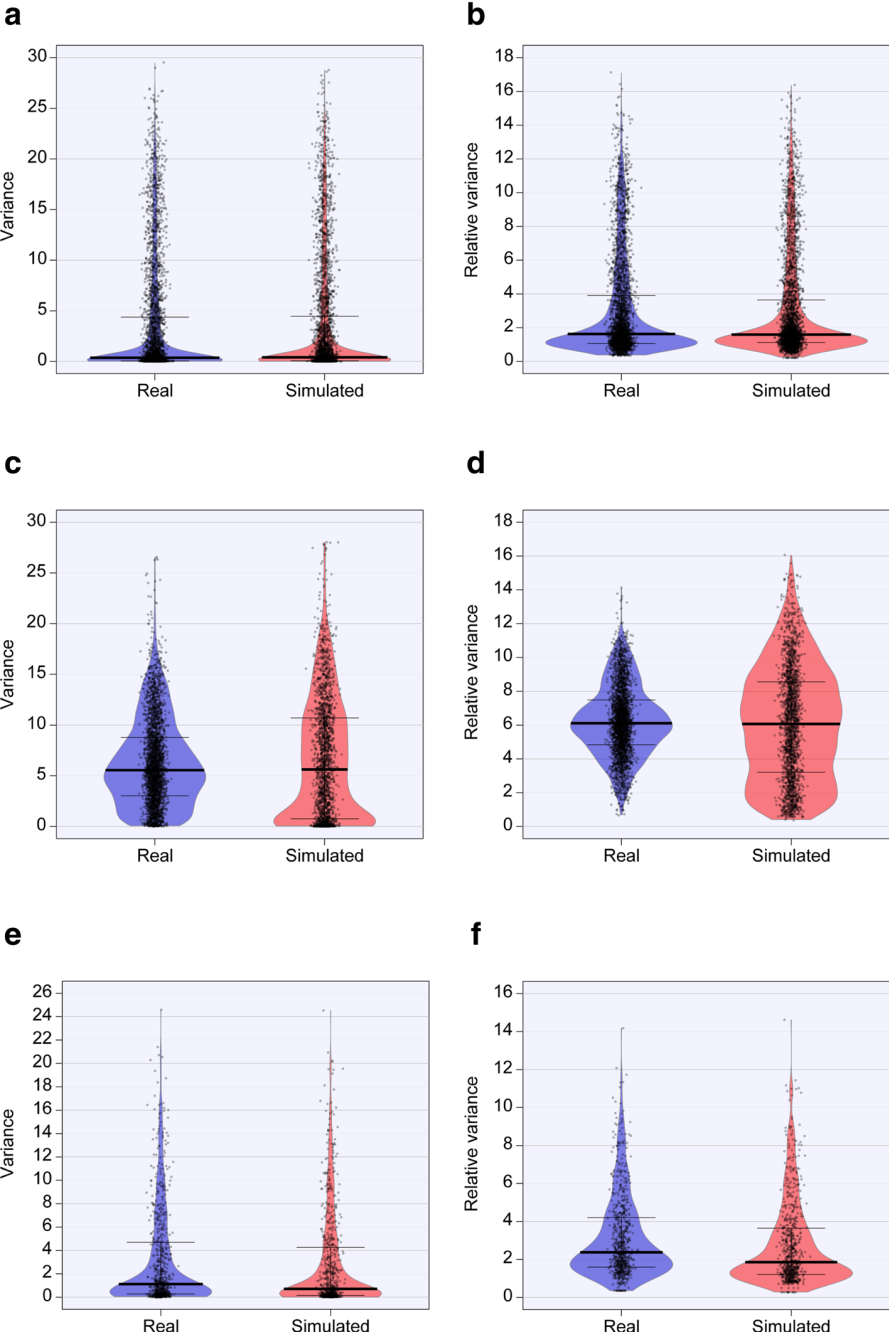
Count variability in real and simulated data. Comparison of Log2 variability values in real and simulated datasets, calculated as variance (**a**, **c**, **e**) and RV (**b**, **d**, **f**). Results are shown for animal gut (first row), raw milk cheese (second row) and HMP (third row) data, excluding zero mean features

**Fig. 6:**
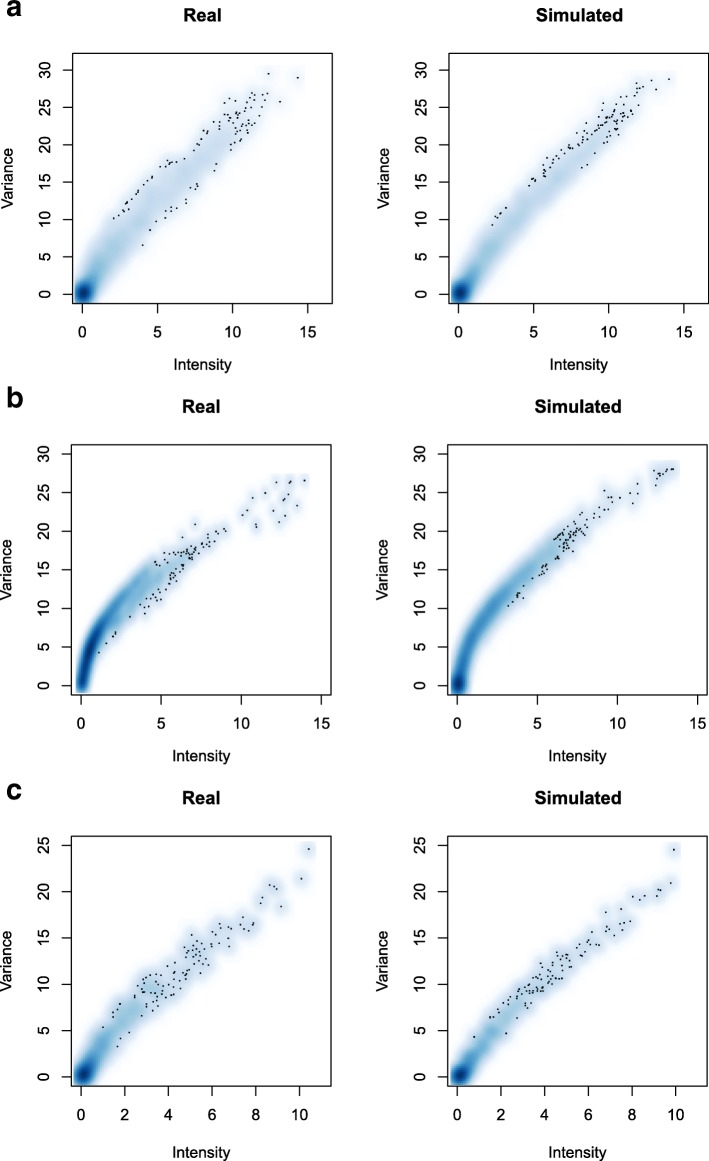
Intensity-variability relation in real and simulated data. Scatter plot of the relation between Log2 count mean intensity and variability in real and simulated datasets, for animal gut (**a**), raw milk cheese (**b**) and HMP (**c**) data, excluding zero mean features

Lastly, the goodness in recreating the dependency between variance and intensity was investigated for all the datasets. The results (Fig. 6) showed a very good adherence to real experimental data characteristics, with animal gut dataset being the one for which we obtained the best results and raw milk cheese dataset being the most problematic one.

## Discussion

Despite a number of different models to simulate 16S rDNA-seq count data were proposed during the last years, no consensus on which one is the most appropriate to use has been reached yet. Additionally, to the best of our knowledge, no simulation tool that underwent adequate validation procedure is currently available to produce synthetic 16S sequencing datasets. Indeed, a lot of different simulation procedures have been proposed in the various banchmarking and new methods presentation papers. However, none of these includes a well-documented, freely accessible and easily usable 16S rDNA-seq count data simulator.

In this work, we propose a gamma-MHG model in which the sequencing process is modelled as a sampling without replacement, i.e. with variable internal probabilities, that follows the rationale of experimental data production and captures directly the mechanisms of structural and random zeros generation. Adopting this modellization, it is possible to consider the compositional nature of 16S rDNA-seq data and to produce sparsity and variability in a natural and intrinsic way, avoiding artificial and possibly imprecise zero count introduction. Indeed, according to this modellization, zero count values rise naturally from the sampling procedure, following the real scenario in which rare OTUs result more frequently than others in zero counts because they are the most probable features not read (i.e. sampled) by the sequencer.

The metaSPARSim performance assessment was performed by considering real OTU tables, estimating the parameters for simulation from them and comparing the resulting simulated count matrices with the real ones, as also done by Zappia et al. in their work [30]. We tested our 16S rDNA-seq data simulator in three very different conditions. This allowed to obtain a solid assessment of performance in different situations.

In particular, the animal gut dataset was characterized by a good number of biological replicates per group (5) and a high but not extreme sparsity level (78.7%). metaSPARSim was able to reconstruct real experimental data properties almost perfectly, in terms of all the three features (sparsity, intensity and variability) considered. In all three scenarios, intensity-sparsity and intensity-variance dependencies, were faithfully reproduced.

Secondly, HMP dataset was chosen to add another challenging element to the testing framework, i.e. a very high biological variability. The biological replicates present in these data were indeed constituted by samples collected in the same body site, but from different individuals and, consequently, they presented a higher variability. The tests confirmed the robustness of the simulator even in this scenario. In fact, simulated sparsity, intensity and variability correctly mimicked the real ones, proving the ability of metaSPARSim in producing realistic human-microbiome-like synthetic dataset.

When the raw milk cheese data were used as metaSPARSim input, a worsening of the performance was observed. We hypothesized that the reason was a poor estimate of the input parameters due to the low number of replicates per experimental group (2 or 3) and the high number of zeros (97.1%). To test this hypothesis, we performed an experiment artificially diminishing the number of replicates and augmenting the number of zeros used in HMP dataset to estimate the input parameters for the simulation. Indeed, the performance of the simulator dropped in terms of ability to reproduce the original dataset. Note however that, in a simulation framework, the purpose is not to create a copy of a real dataset, but rather to generate a realistic count table from a known given input.

A possible limitation of this work is the lack of comparison with other simulation approaches proposed in the literature. However, as said above, the availability of simulators for synthetic count data generation is limited to papers that use simulators to benchmark new proposed analysis methods or literature methods. Most of the times, a software is not available, but only the R scripts used to perform the simulations are released with poor guidelines about how to use them. In addition, in order to compare the resulting simulated count matrices with the real ones, a simulator should accept as input real OTU tables and estimate the parameters for the simulation from them. For the above reasons, a direct comparison with other software tools was not possible within this work.

## Conclusions

The availability of count data simulators is extremely valuable for methods developers, which can exploit the ground truth provided by simulated data to test and validate their tools. In addition, simulated data are useful even for end users who want to find the most accurate analysis methods fitting their dataset characteristics among the many available in the literature. Indeed, the availability of simulated data allows to assess state of the art analysis tools and to identify the more suitable one for the specific scenario. Thus, we believe that metaSPARSim could be a valuable tool for researchers involved in developing, testing and using robust and reliable data analysis methods in the context of 16S rDNA-seq.

## Additional file

Additional file 1 Supplementary material, including Supplementary Figures and Tables. (PDF 8514 kb)

## Data Availability

metaSPARSim is available at http://sysbiobig.dei.unipd.it/?q=Software#metaSPARSimand https://gitlab.com/sysbiobig/metasparsimunder GNU General Public License. The other datasets generated and analyzed during the current study are available from the corresponding author on reasonable request.

## Abbreviations

HMP: Human microbiome project
HTS: High-throughput sequencing
MHG: Multivariate hypergeometric
NB: Negative binomial
NBH: Negative binomial hurdle
NGS: Next generation sequencing
OTU: Operational taxonomic unit
PCR: Polymerase chain reaction
PH: Poisson hurdle
RDI: Raw (data), description and inference
ZI: Zero-inflated
ZINB: Zero-inflated negative binomial
ZIP: Zero-inflated Poisson
