# Supplementary material for: metaSPARSim: a 16S rRNA gene sequencing count data simulator
Source: BMC Bioinformatics. 2019 Nov 22;20(Suppl 9):416. doi: 10.1186/s12859-019-2882-6 (PMC6873395; doi:10.1186/s12859-019-2882-6)
Supplement: Supplementary file 1 — Supplementary material, including Supplementary Figures and Tables. (PDF 8514 kb) [file 12859_2019_2882_MOESM1_ESM.pdf]

Parameter estimation from real datasets

The estimation procedure implemented in metaSPARSim is composed by single modules, all singularly accessible for specific use. More precisely:

- `estimate_intensity` function: performs the estimate of intensity vectors to give metaSPARSim as input. It summarizes biological replicates information through the application of an aggregation function (mean, median or maximum) chosen by the user, that can also decide whether to consider or discard null values during this procedure.

- `estimate_variability` function: performs the estimate of replicates variability within a group for each OTU calculating the dispersion parameter as in *edgeR* [1].

- `estimate_library_size` function: extracts real library sizes from the original dataset.

However, for the estimation of the complete parameter set, function *estimate_parameters_from_data* has been implemented as a wrapper of the three above functions.

Presets

Here is described the set of pre-computed parameters for 16S count matrix simulation included in metaSPARSim. The parameter sets collected in metaSPARSim have been estimated using metaSPARSim from five different real data, describing more than 1100 samples across almost 90 different sample groups. These datasets were chosen to represent 16S rDNA sequencing data diversity, having all of them peculiar characteristics in terms of number of samples, constitutive groups, sequencing depths and sample source. The first two sets were estimated from data coming from experiments performed in the context of this work (*R1-R2* sets), while the other ones are estimated from Human Microbiome Project (HMP) [2, 3] data (*R3-R4* sets) and Atacama soil [4] data (*R5*).

Real data

In this section are described in detail the two proprietary real datasets used for simulator testing.

Animal gut microbiome This sample collection had the aim of monitoring chicken gut microbiota modifications in the first 4 weeks of life in occurrence of *Campylobacter* spp. infection, and comparing them with healthy chickens risen in analogous conditions but within farms in which no *Campylobacter* spp. infection occurred. For this study, we selected four broiler farms belonging to the same supply chain, half of which became positive for *Campylobacter* spp. during the sampling period, while the other half showed no infection during all the 4 weeks of monitoring. Five samples were collected from each farm at 5 different time points (7th, 14th, 18th, 21th and 28th day of chickens' age), for a total of 110 caecal samples. For the two positive farms, ten samples (five positive and five negative for *Campylobacter* spp.) were collected for the time points in which infection was first detected. A scheme of this experiment is reported in Figure 1. Total DNA was then extracted and V3-V4 regions of 16S rRNA gene were amplified with the primers CCTACGGGNGGCWGCAG (forward) and GACTACHVGGGTATCTAATCC (reverse), following Klindworth et al. [5], and sequenced on HiSeq2500 platform in RAPID mode (2x250 bp). Sequencing data underwent a quality control procedure using the FastQC tool [6]. Data were then cleaned by removing adapters, primers and performing dereplication of sequences using a in-house bash script. In addition, data were filtered based on the quality and length of the reads, so that only data with a quality higher than a given threshold (QPhred ≥ 20) and reads whose length exceeded 100bp were retained. All subsequent steps were performed using QIIME1 [7] pipeline (version 1.9.0). Data obtained from the filtering step underwent read pairing, in order to obtain a single file in which the reads obtained by sequencing the 16S fragments on the forward strand and on the reverse are joined by their overlapping region. Then, OTU picking step was performed, assigning reads to a particular taxonomy by directly mapping the same reads to a 16S sequences database (GreenGenes database [8], last release May 2013).

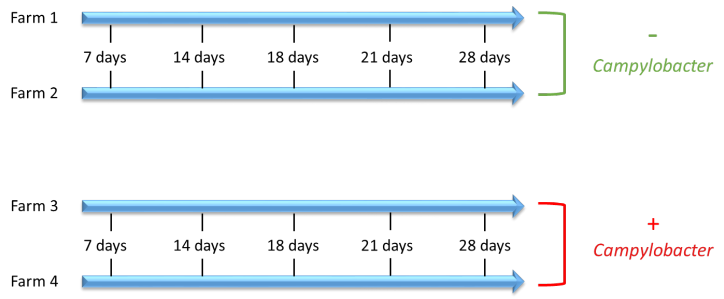

Figure 1 Scheme representing the experimental design of animal gut microbiome study.

Food microbiome from raw milk cheese The second study had the

aim of following the dynamics of the microbial community of "Latteria" raw milk cheese during its ripening period, in natural ageing conditions and in presence of contamination by pathogens like *Listeria monocytogenes* and *Staphilococcus aureus*. The cheesemaking was made in a dairy in Friuli Venezia Giulia region, following all the typical steps of this particular raw milk cheese production. The design was made by 4 types of cheesemaking:

- plain, with no contamination
- contaminated with *Listeria innocua*, chosen for security reasons as a substitute of *Listeria monocytogenes* as being characterized by the same dynamical behaviour
- contaminated with *Staphilococcus aureus*
- contaminated with both *Listeria innocua* and *Staphilococcus aureus*.

Each cheesemaking was performed in triple replicate, to access biological variability. Samples from from cheese till the 30rd day of ripening period were collected, for a total of 10 sampling time points and 120 samples (12 from cheese at each time point). A scheme of this experiment is showed in Figure 2. Total RNA was extracted and retrotranscribed; then V3-V4 regions of 16S rRNA gene were amplified with the primers CCTACGGGNGGCWGCAG (forward) and GACTACHVGGGTATCTAATCC (reverse), following Klindworth et al. [5], and sequenced on HiSeq2500 platform in RAPID mode (2x250 bp). After sequencing, a control procedure using the FastQC tool [6] was performed on resultant data. After that, QIIME2 [7] pipeline (version 2017.11) was used to perform sequence quality control and feature table construction via DADA2 pipeline, which also performs phiX reads and chimeric sequences filtering. Taxonomic assignment was obtained using the QIIME2 Naive Bayes classifier pre-trained on Silva [9] database.

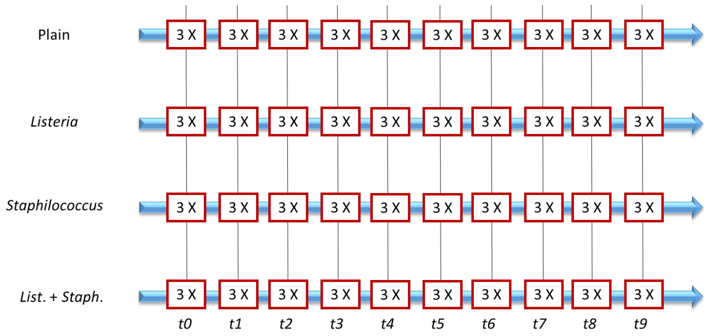

Figure 2 Scheme representing the experimental design of animal gut microbiome study.

Human Microbiome Project data Data obtained in the context of the Human Microbiome Project (HMP) ([2, 3]) were used to test for metaSPARSim performance. The project had the aim of creating resources to easily characterize the human microbiota. Within this project, the microbial communities from 300 healthy individuals across several different sites on the human body was characterized: nasal passages, oral cavity, skin, gastrointestinal tract, and urogenital tract samples were collected and variable regions 3-5 (V35) of 16S rRNA gene were sequences (Figure 3). The two datasets considered in this work that were derived from HMP data are composed by a subsample of this publicly available dataset (`https://qiita.ucsd.edu/study/description/1928`), taking 5 random samples from 8 sampled groups belonging to the oral cavity part of data: hard palate, gingivae, tongue dorsum, teeth, palatine tonsils, throat, saliva and buccal mucosa. Samples were randomly drawn from the available.

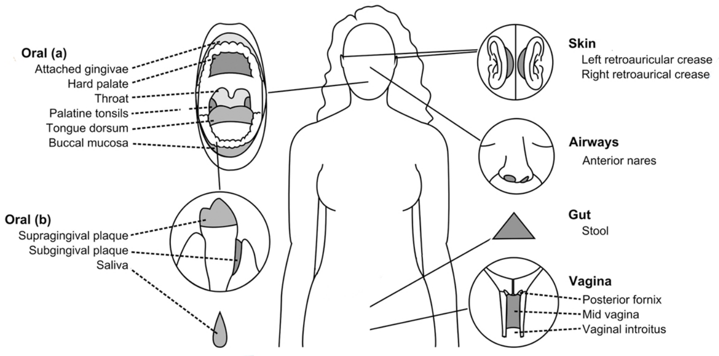

Figure 3 Human Microbiome Project sampling map. Adapted from [10]

Evaluation metrics for metaSPARSim performance assessment

The goodness in reproducing realistic sparsity, intensity and variability characteristics was evaluated following different both qualitative and quantitative measures:

- **Q–Q (quantile-quantile) plots** [11]: a graphical method for comparing two probability distributions by plotting their quantiles against each other. The two compared distributions are considered to be equal when plotted data lay on the diagonal.
- **boxplots** [12]: a method for graphically representing numerical data collections through their quartiles.

- **RDI (Raw data, Descriptive statistics, and Inferential statistics) plots** [13]: this kind of plots permit to represent both punctual and distribution information, joining scatter plot, box plot and density plot together.

- **Mann-Whitney U test** [14]: applied to relative abundances vectors. This non parametric test is based on the null hypothesis that the two tested samples come from the same population (i.e. they both have the same median). If the resultant P-value is less than a fixed significance level (here 0.05), then the null hypothesis is rejected in favour of the alternative hypothesis, i.e. the two samples come from different populations. This test was used to check for possible statistically significant dissimilarities between real and simulated vectors of intensities and variances.

- **Cohen's *d*** [15]: applied to relative abundances vectors. It is one measure associated with the calculation of so-called effect size, a quantitative measure of the magnitude of a phenomenon; here, the effect size quantifies the size of the difference between two groups. In this analysis, we decided to include it alongside the significance test because it has been widely shown ([16],[17],[18]) that when examining effects using large samples significant testing can be misleading because even small or trivial effects are likely to produce statistically significant results. Thus, reporting only the significant P-value for an analysis is not adequate to fully understand the results. Cohen's d is defined as the difference between two means divided by a standard deviation for the data, i.e.
$$d = \frac{\mu_1 - \mu_2}{s}, \tag{1}$$
where s is the pooled standard deviation, defined as
$$s = \sqrt{\frac{(n_1 - 1)s_1^2 + (n_2 - 1)s_2^2}{n_1 + n_2 - 2}} \tag{2}$$
where n_1 and n_2 are the two sample sizes and s_1 and s_2 are the related standard deviations. Table 1 contains cut-offs for magnitudes interpretation, as initially suggested by Cohen and expanded by Sawilowsky[19].

Supplementary Figures and Tables

Table 1 Cohen's d cut-offs.

Effect size	d	Reference
Negligible	< 0.01	Sawilowsky, 2009
Very small	0.01	Sawilowsky, 2009
Small	0.20	Cohen, 1988
Medium	0.50	Cohen, 1988
Large	0.80	Cohen, 1988
Very large	1.20	Sawilowsky, 2009
Huge	2.0	Sawilowsky, 2009

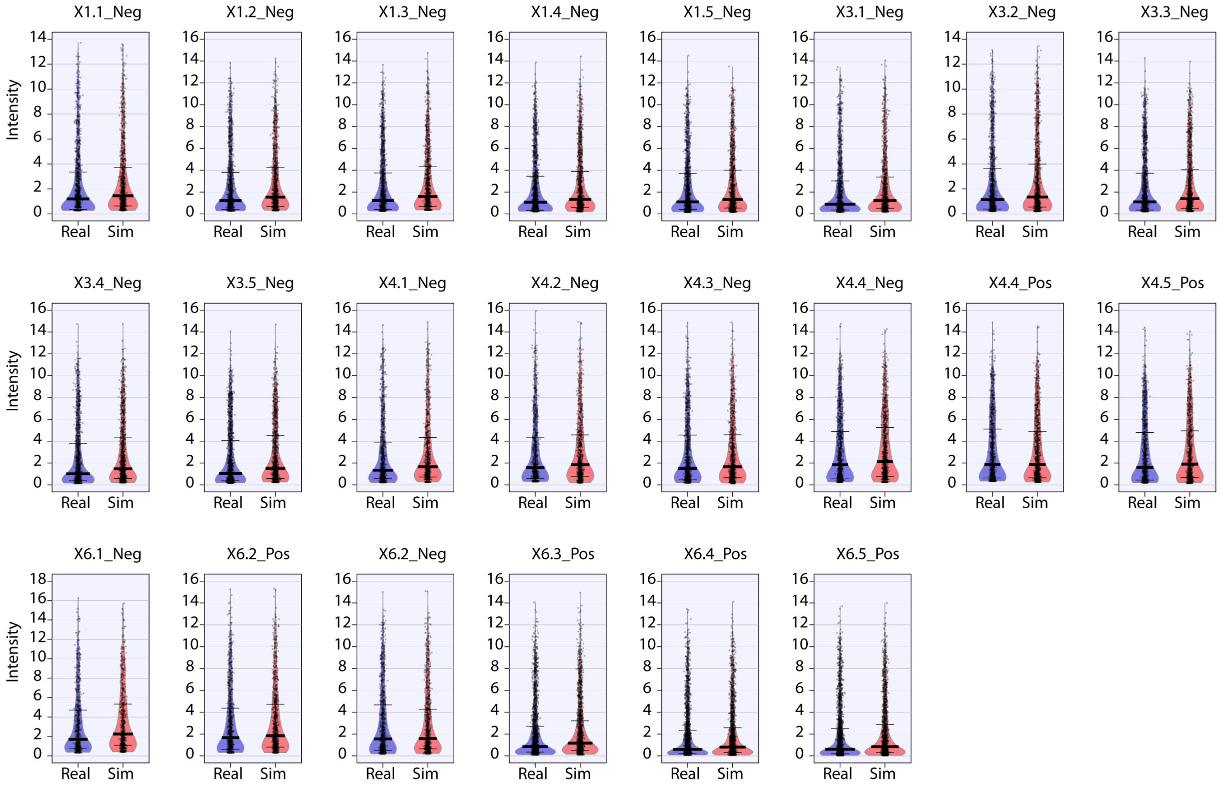

Figure 4 RDI plots of Log2 count values of real and simulated data within each group for animal gut dataset, excluding zero mean features.

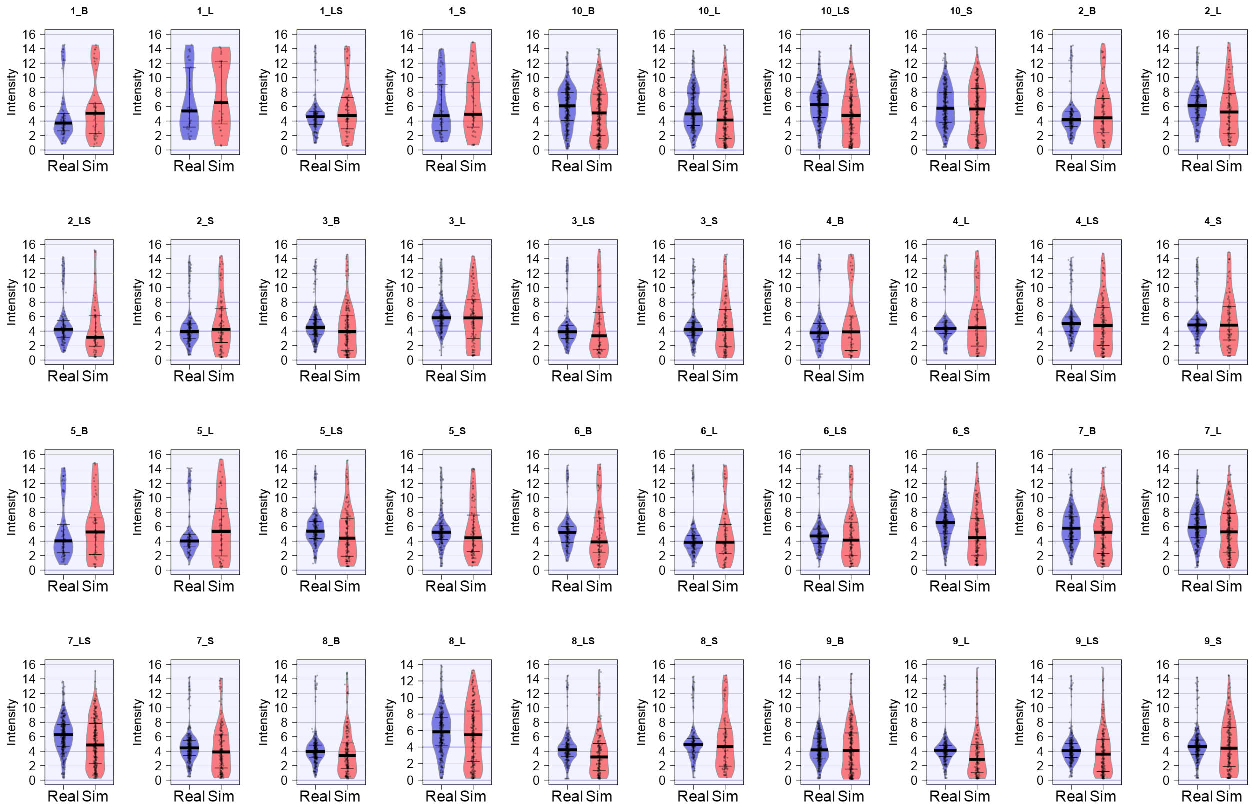

Figure 5 RDI plots of Log2 count values of real and simulated data within each group for raw milk cheese dataset, excluding zero mean features.

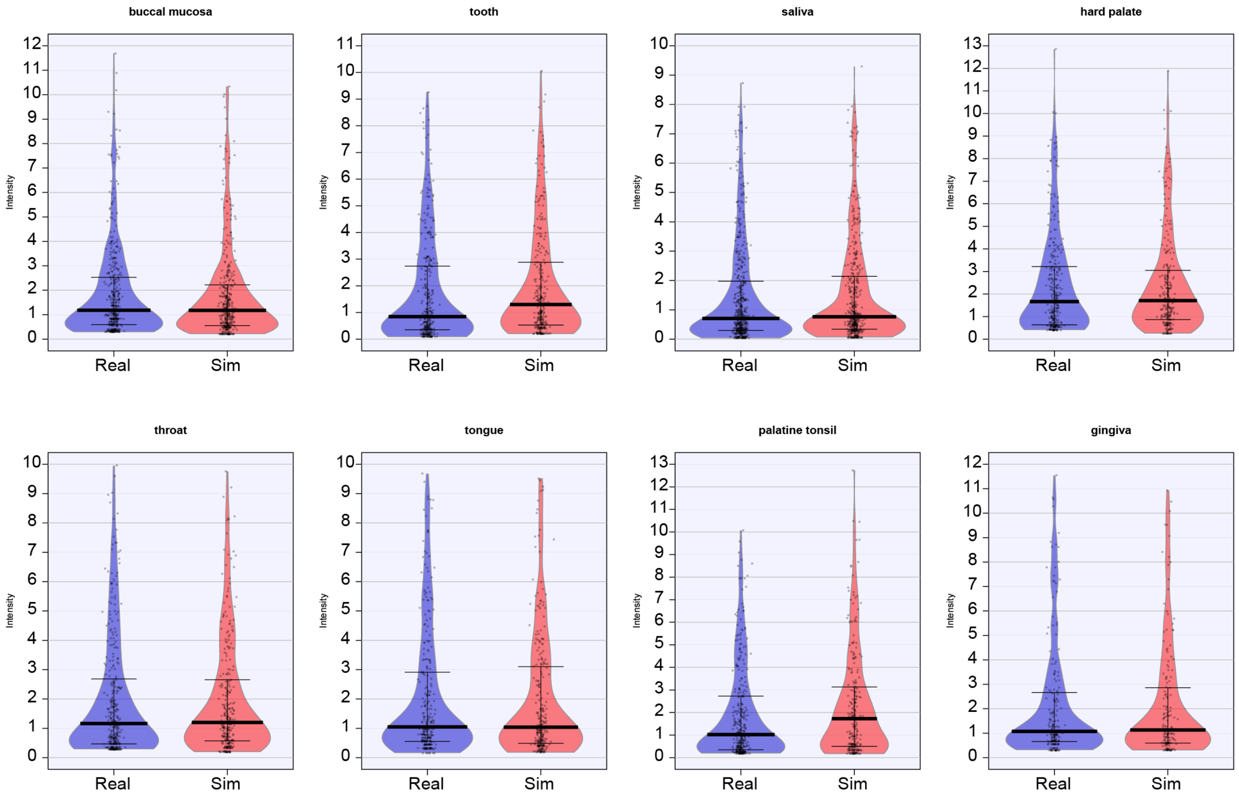

Figure 6 RDI plots of Log2 count values of real and simulated data within each group for HMP dataset, excluding zero mean features.

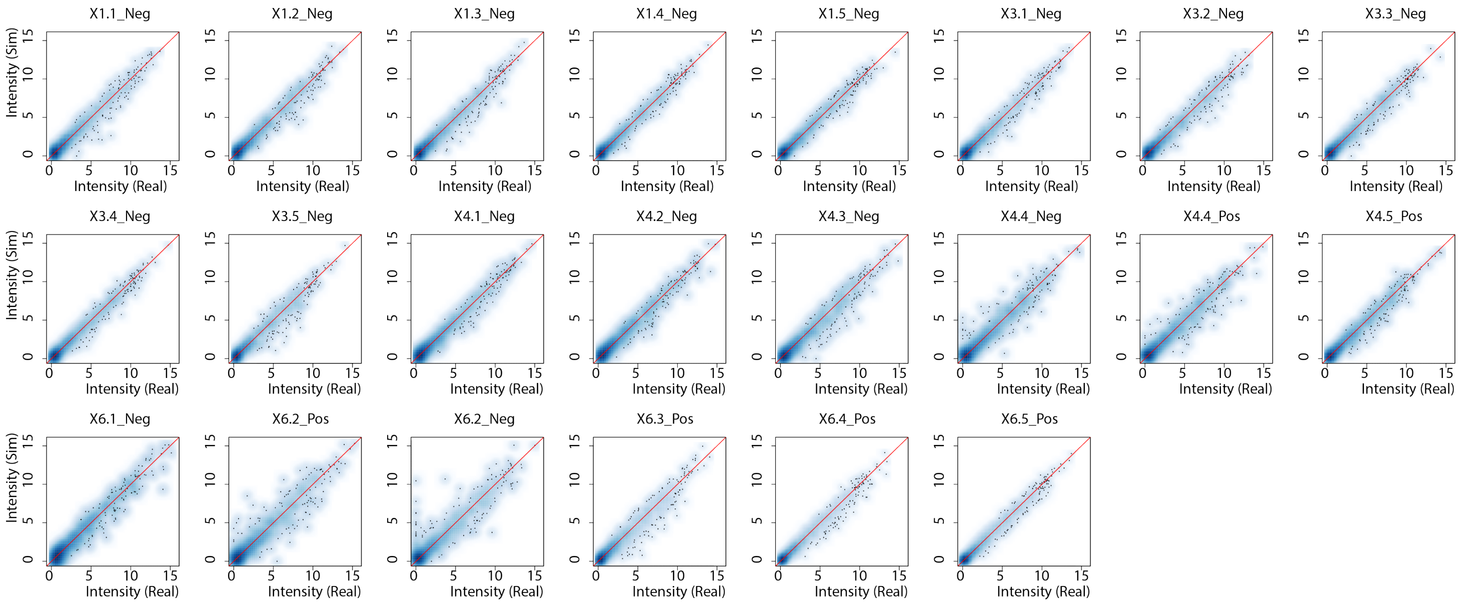

Figure 7 Scatter plots of Log2 count values of real and simulated data within each group for animal gut dataset, excluding cases with zero counts in both data.

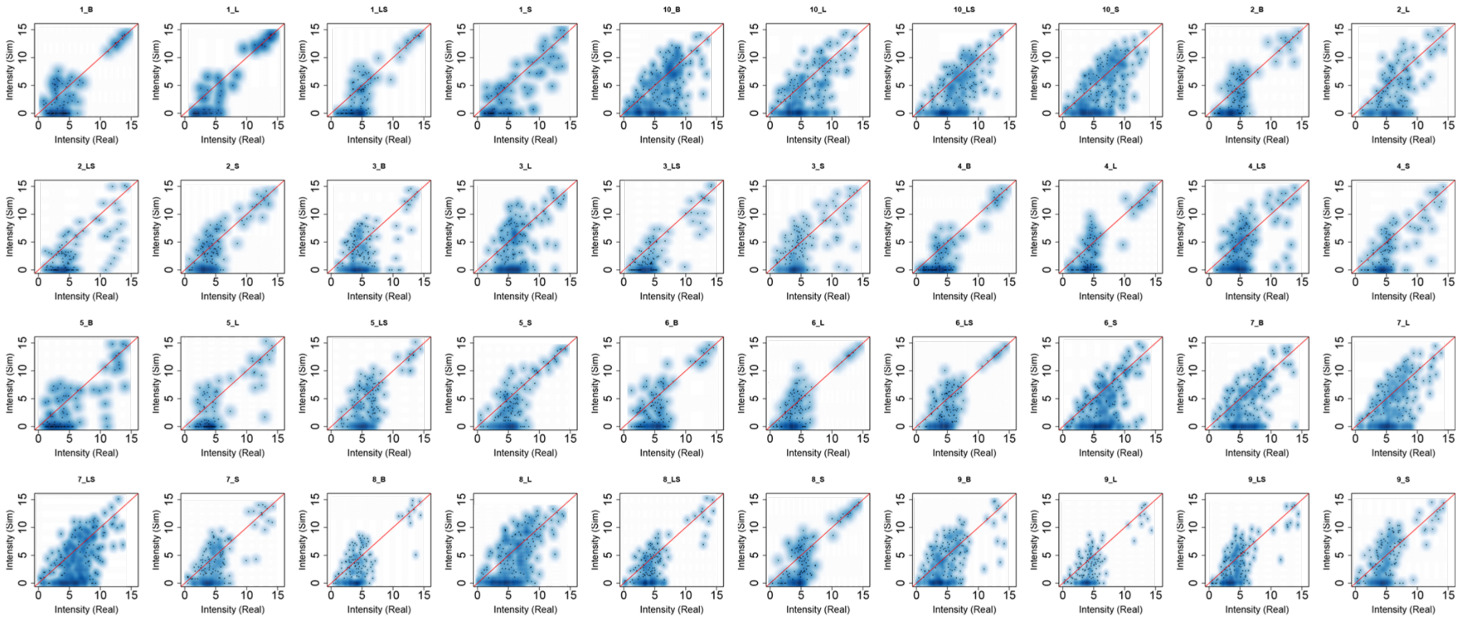

Figure 8 Scatter plots of Log2 count values of real and simulated data within each group for raw milk cheese dataset, excluding cases with zero counts in both data.

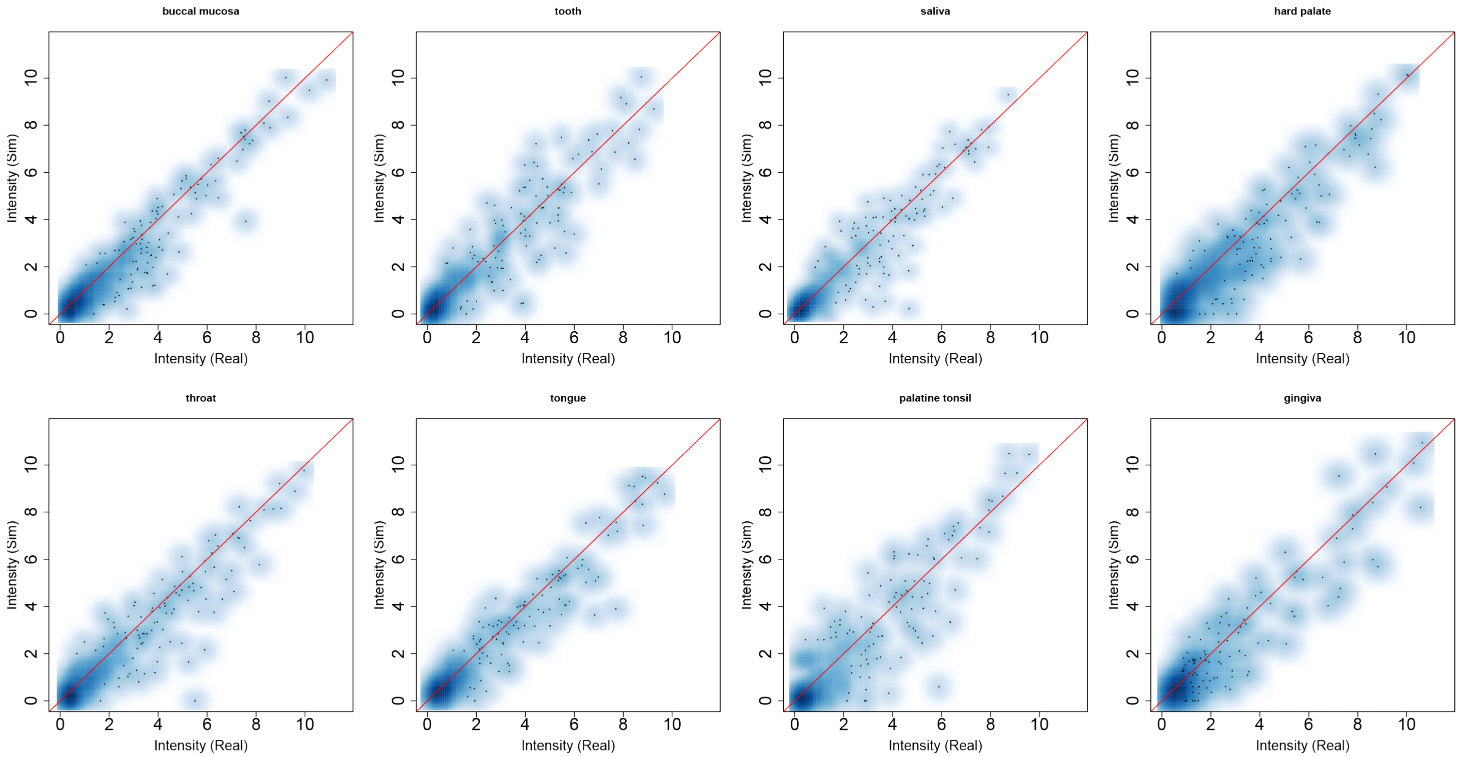

Figure 9 Scatter plots of Log2 count values of real and simulated data within each group for HMP dataset, excluding cases with zero counts in both data.

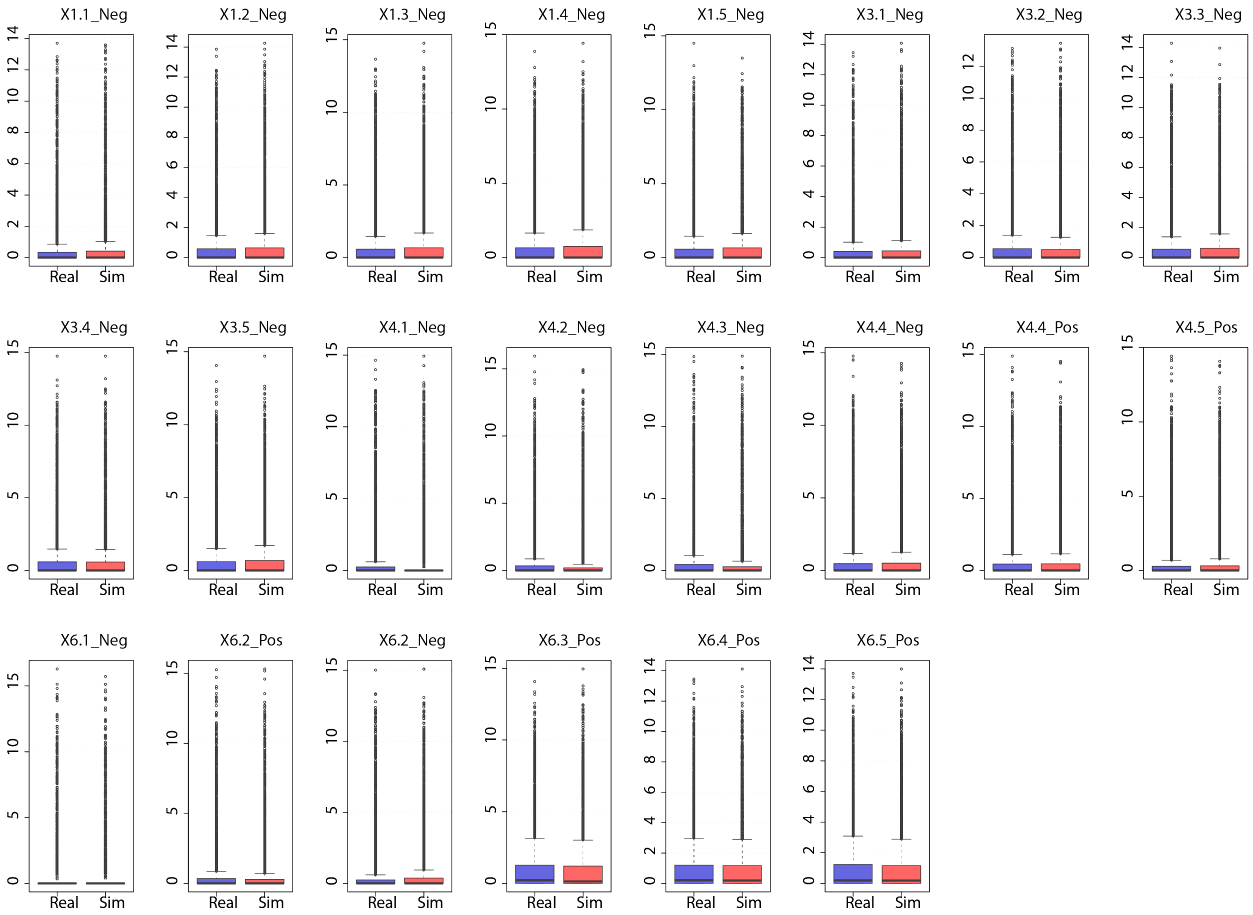

Figure 10 Box plots of Log2 count values of real and simulated data within each group for animal gut dataset.

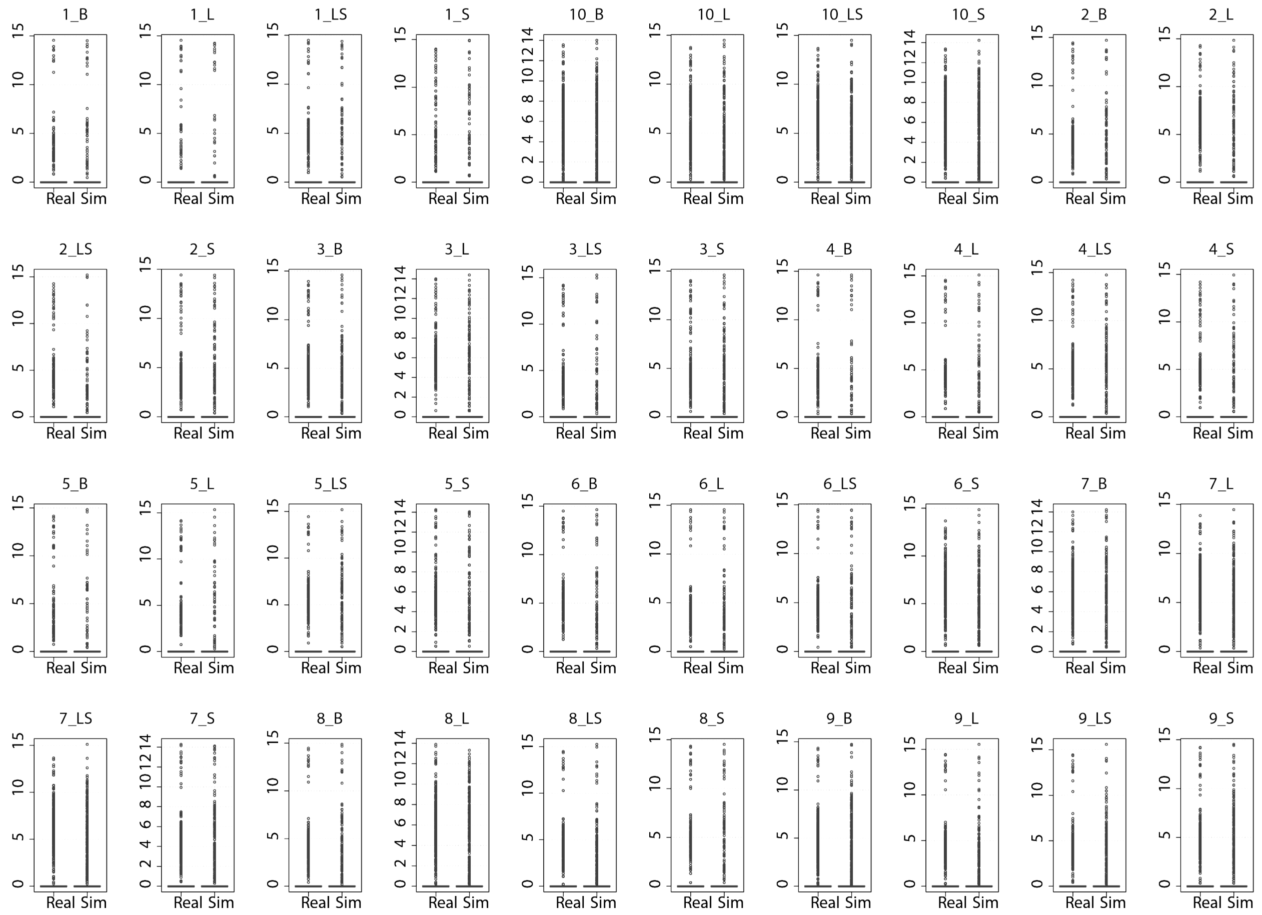

Figure 11 Box plots of Log2 count values of real and simulated data within each group for raw milk cheese dataset.

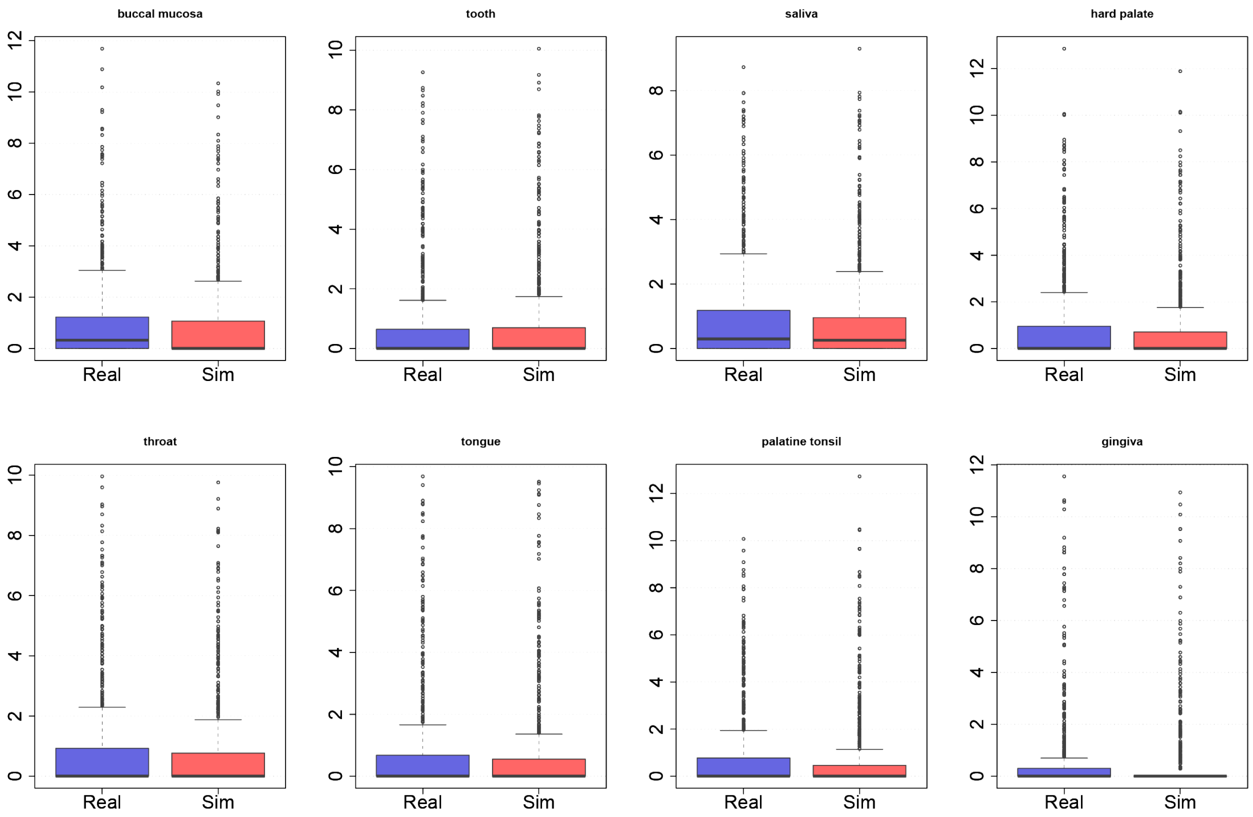

Figure 12 Box plots of Log2 count values of real and simulated data within each group for HMP dataset.

Table 2 Mann-Whitney U tests and effect size results in comparing real and simulated mean count distribution within groups for animal gut dataset.

Group	P value	Significance	Cohen's d magnitude	Bootstrap significance (%)
X1.1_Neg	0.039	Y	Negligible	0
X1.2_Neg	0.027	Y	Negligible	0
X1.3_Neg	0.001	Y	Negligible	0
X1.4_Neg	0.013	Y	Negligible	0
X1.5_Neg	0.109	N	− − −	0
X3.1_Neg	0.002	Y	Negligible	0
X3.2_Neg	0.013	Y	Negligible	0
X3.3_Neg	0.013	Y	Negligible	0
X3.4_Neg	0.002	Y	Negligible	0
X3.5_Neg	0.004	Y	Negligible	0
X4.1_Neg	0.009	Y	Negligible	0
X4.2_Neg	0.133	N	− − −	0
X4.3_Neg	0.02	Y	Negligible	0
X4.4_Neg	0.373	N	− − −	0
X4.4_Pos	0.757	N	− − −	0
X4.5_Pos	0.065	N	− − −	0
X6.1_Neg	0.020	Y	Negligible	0
X6.2_Pos	0.194	N	− − −	0
X6.2_Neg	0.179	N	− − −	0
X6.3_Pos	0.001	Y	Negligible	0
X6.4_Pos	0.006	Y	Negligible	0
X6.5_Pos	0.002	Y	Negligible	0

Table 3 Mann-Whitney U tests and effect size results in comparing real and simulated mean count distribution within groups for raw milk cheese dataset.

Group	P value	Significance	Cohen's d magnitude	Bootstrap significance (%)
1_B	0.004	Y	Negligible	0.88
1_L	0.111	N	− − −	0.10
1_LS	0	Y	Negligible	5.36
1_S	0.008	Y	Negligible	0.48
10_B	0.001	Y	Negligible	0.20
10_L	0	Y	Negligible	1.96
10_LS	0	Y	Negligible	1.09
10_S	0	Y	Negligible	0.58
2_B	0	Y	Negligible	2.34
2_L	0	Y	Negligible	6.32
2_LS	0	Y	Negligible	3.86
2_S	0	Y	Negligible	1.35
3_B	0	Y	Negligible	8.89
3_L	0	Y	Negligible	2.99
3_LS	0	Y	Negligible	8.01
3_S	0	Y	Negligible	4.33
4_B	0	Y	Negligible	5.38
4_L	0.003	Y	Negligible	0.53
4_LS	0.002	Y	Negligible	0.35
4_S	0	Y	Negligible	3.58
5_B	0.005	Y	Negligible	0.83
5_L	0	Y	Negligible	1.94
5_LS	0	Y	Negligible	1.29
5_S	0	Y	Negligible	4.00
6_B	0	Y	Negligible	1.81
6_L	0	Y	Negligible	2.60
6_LS	0	Y	Negligible	2.15
6_S	0	Y	Negligible	2.02
7_B	0	Y	Negligible	7.15
7_L	0	Y	Negligible	3.99
7_LS	0.001	Y	Negligible	0.19
7_S	0	Y	Negligible	1.97
8_B	0	Y	Negligible	4.69
8_L	0	Y	Negligible	0.61
8_LS	0	Y	Negligible	0.91
8_S	0.002	Y	Negligible	0.60
9_B	0	Y	Negligible	0.51
9_L	0	Y	Negligible	4.28
9_LS	0.002	Y	Negligible	0.16
9_S	0	Y	Negligible	0.90

Table 4 Mann-Whitney U tests and effect size results in comparing real and simulated mean count distribution within groups for HMP dataset.

Group	***P*** **value**	**Significance**	**Cohen's *d* magnitude**	**Bootstrap significance (%)**
buccal mucosa	0.016	Y	Negligible	0
tooth	0.056	N	− − −	0.01
saliva	0.052	N	− − −	0
hard palate	0.016	Y	Negligible	0.01
throat	0.024	Y	Negligible	0
tongue	0.228	N	− − −	0
palatine tonsil	0	Y	Negligible	0.63
gingiva	0.141	N	− − −	0

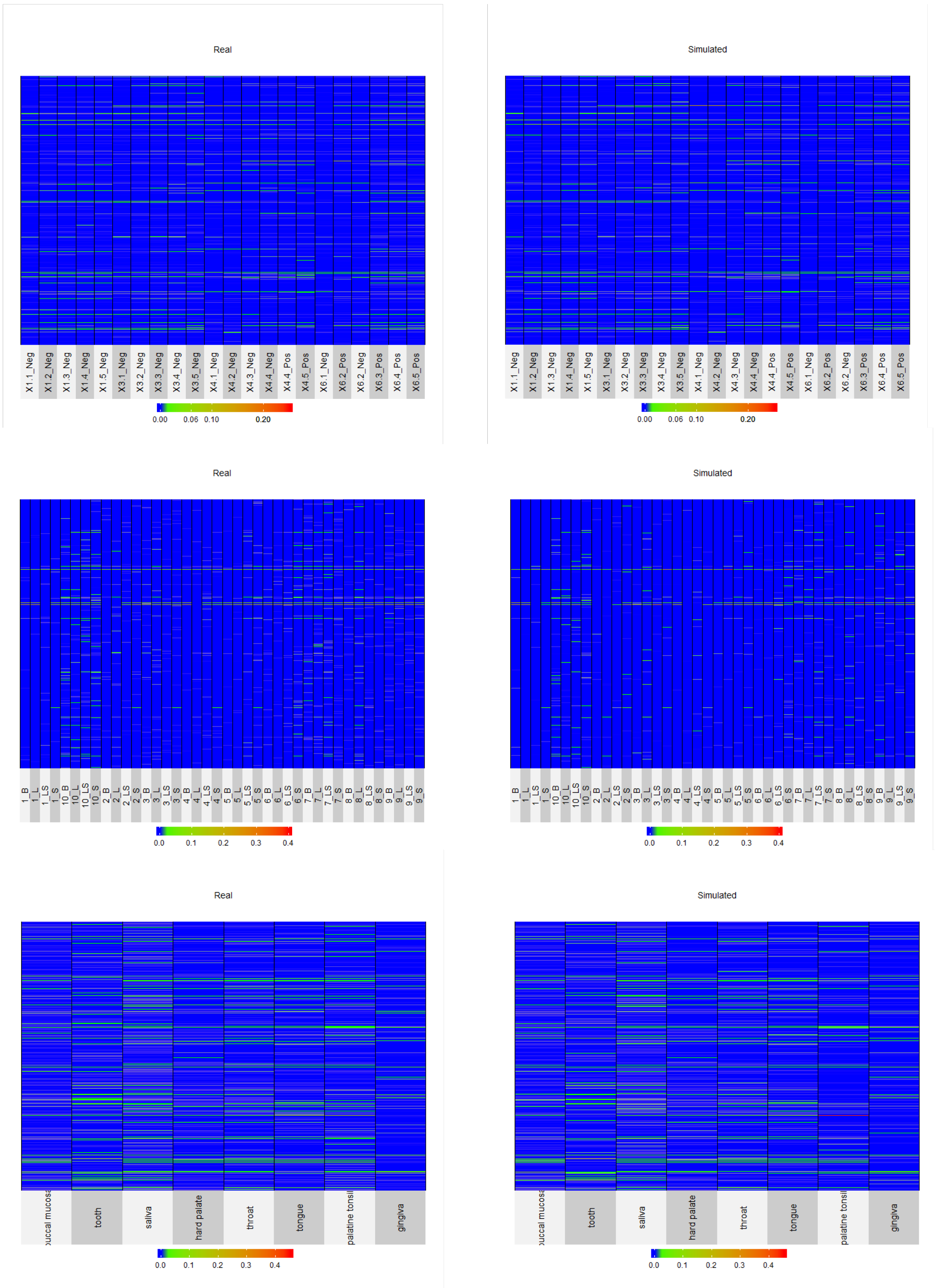

Figure 13 Heatmaps of proportional abundances of real (left panels) and simulated (right panels) datasets. Results are shown for animal gut (first row), raw milk cheese (second row) and HMP (third row) data.

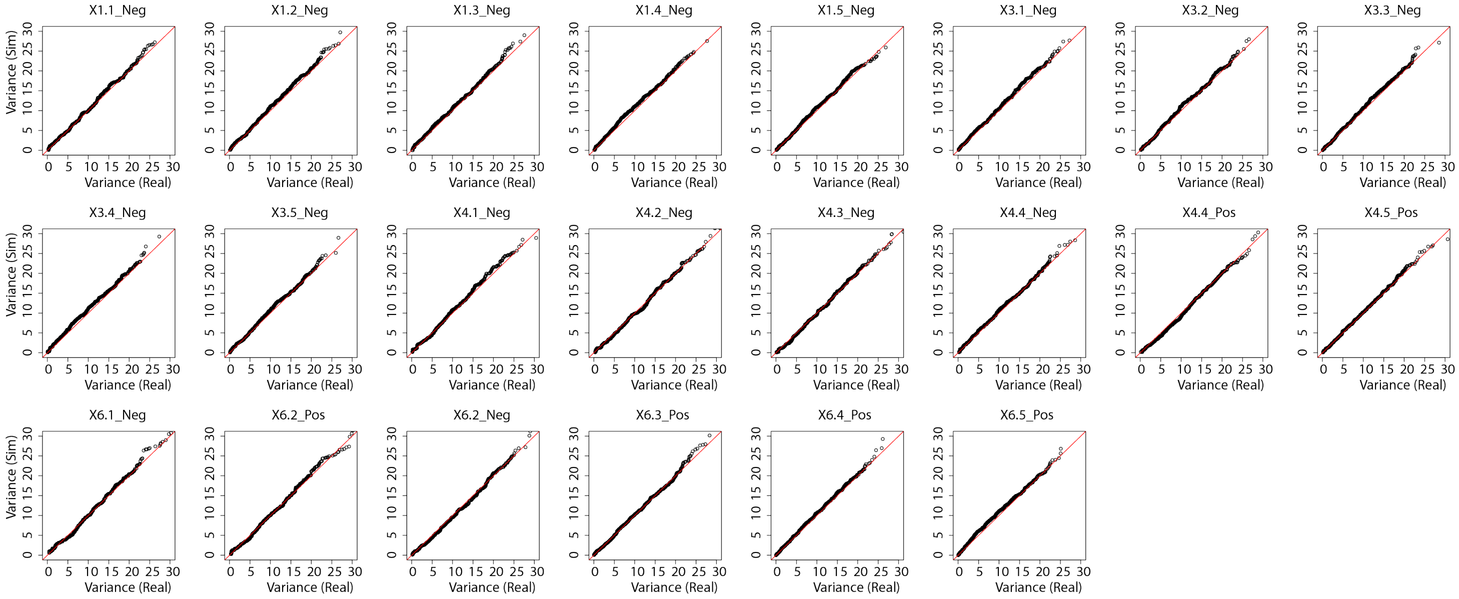

Figure 14 Q−Q plots of of Log2 variance values in real and simulated datasets within each group for animal gut dataset, excluding zero mean features.

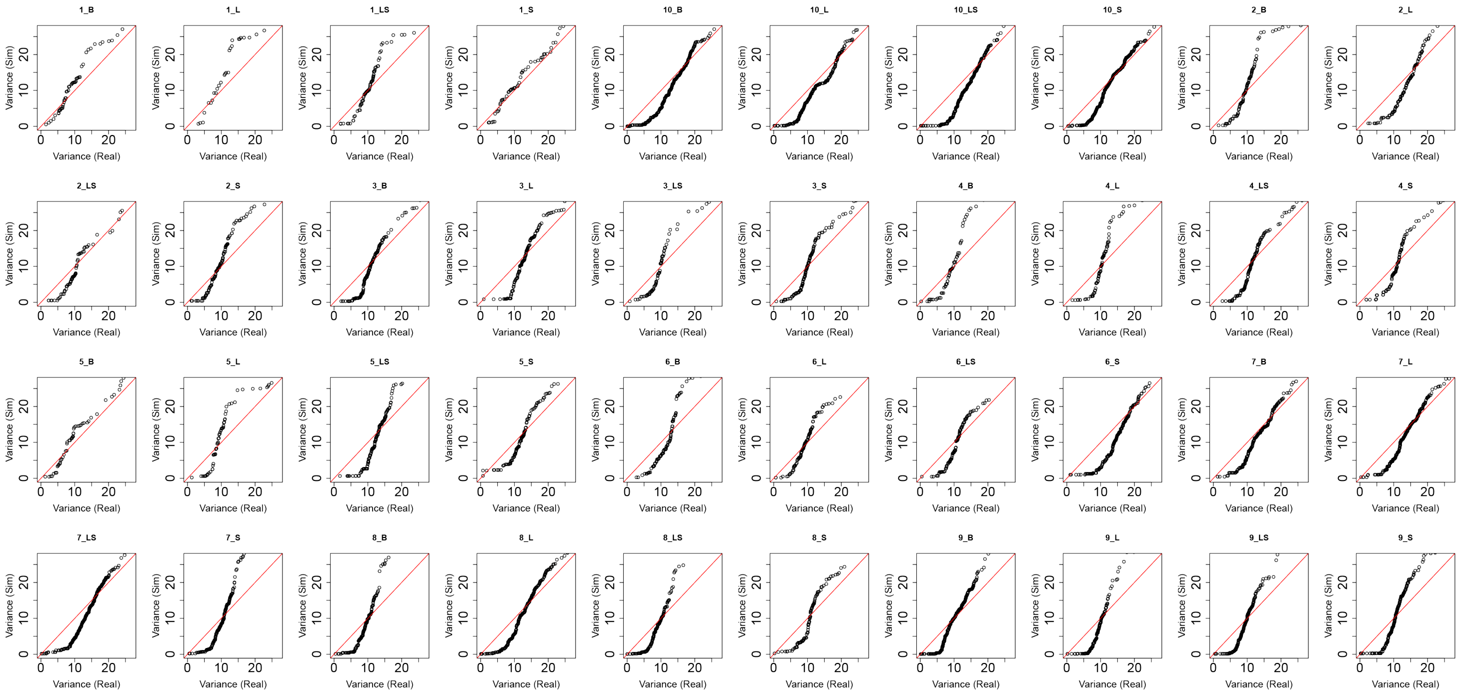

Figure 15 Q−Q plots of of Log2 variance values in real and simulated datasets within each group for raw milk cheese dataset, excluding zero mean features.

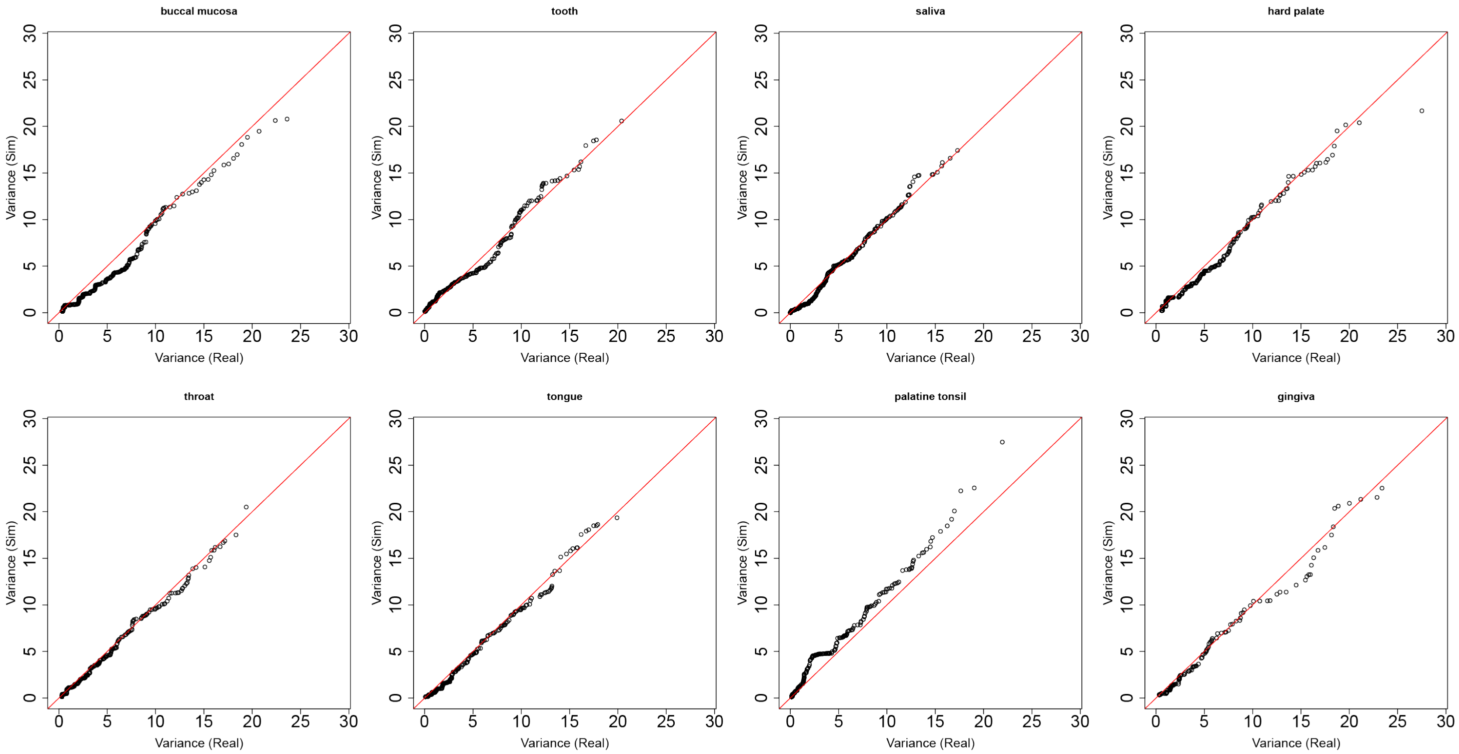

Figure 16 Q−Q plots of of Log2 variance values in real and simulated datasets within each group for HMP dataset, excluding zero mean features.

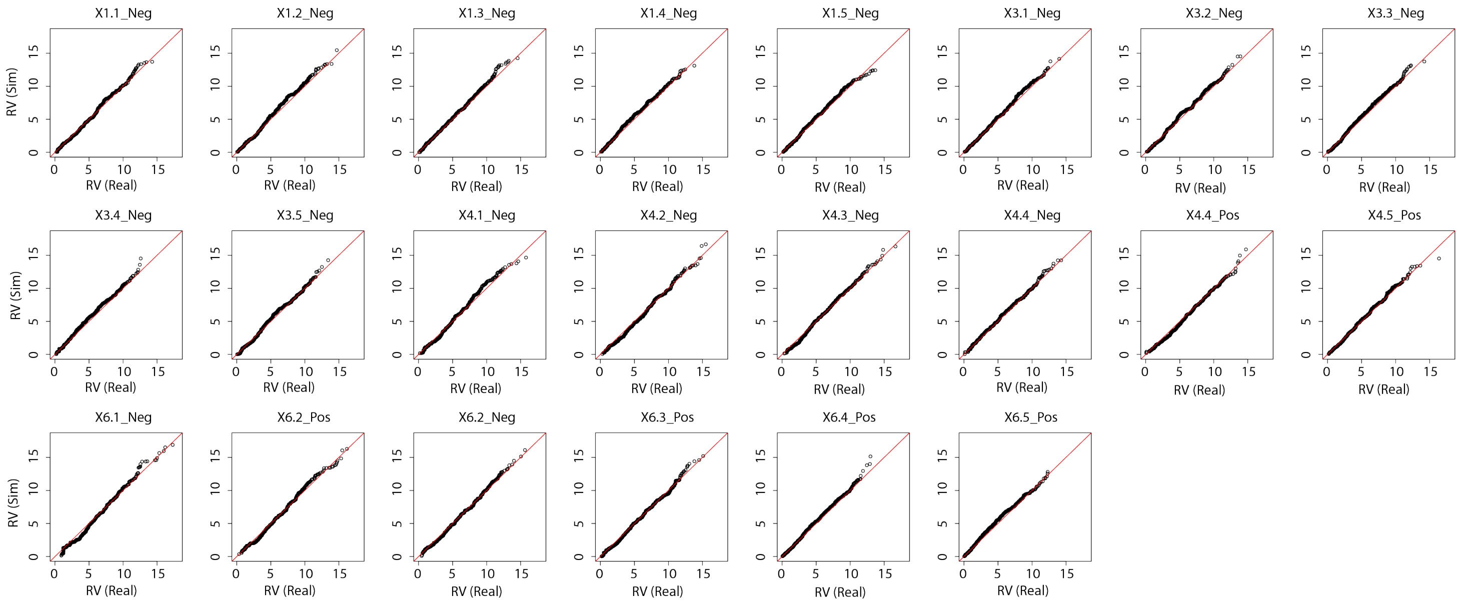

Figure 17 Q−Q plots of of Log2 RV values in real and simulated datasets within each group for animal gut dataset, excluding zero mean features.

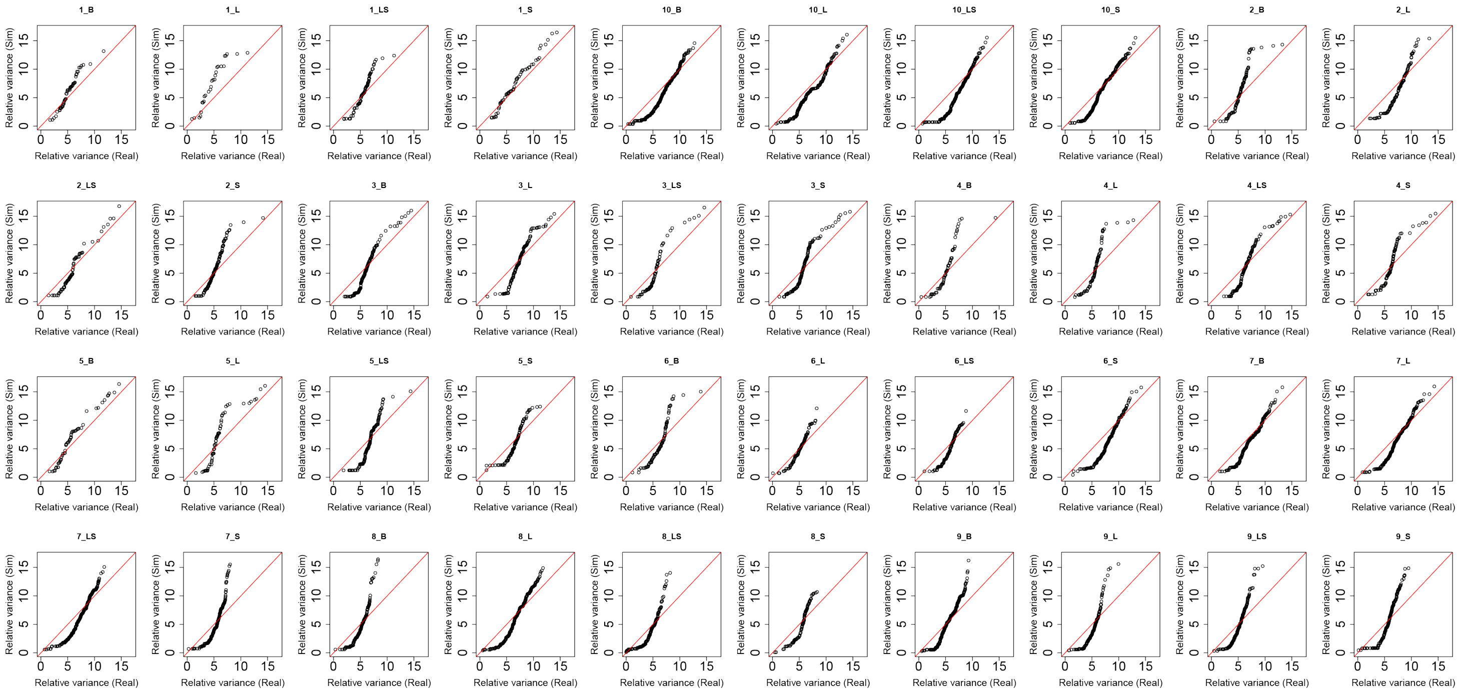

Figure 18 Q−Q plots of of Log2 RV values in real and simulated datasets within each group for raw milk cheese dataset, excluding zero mean features.

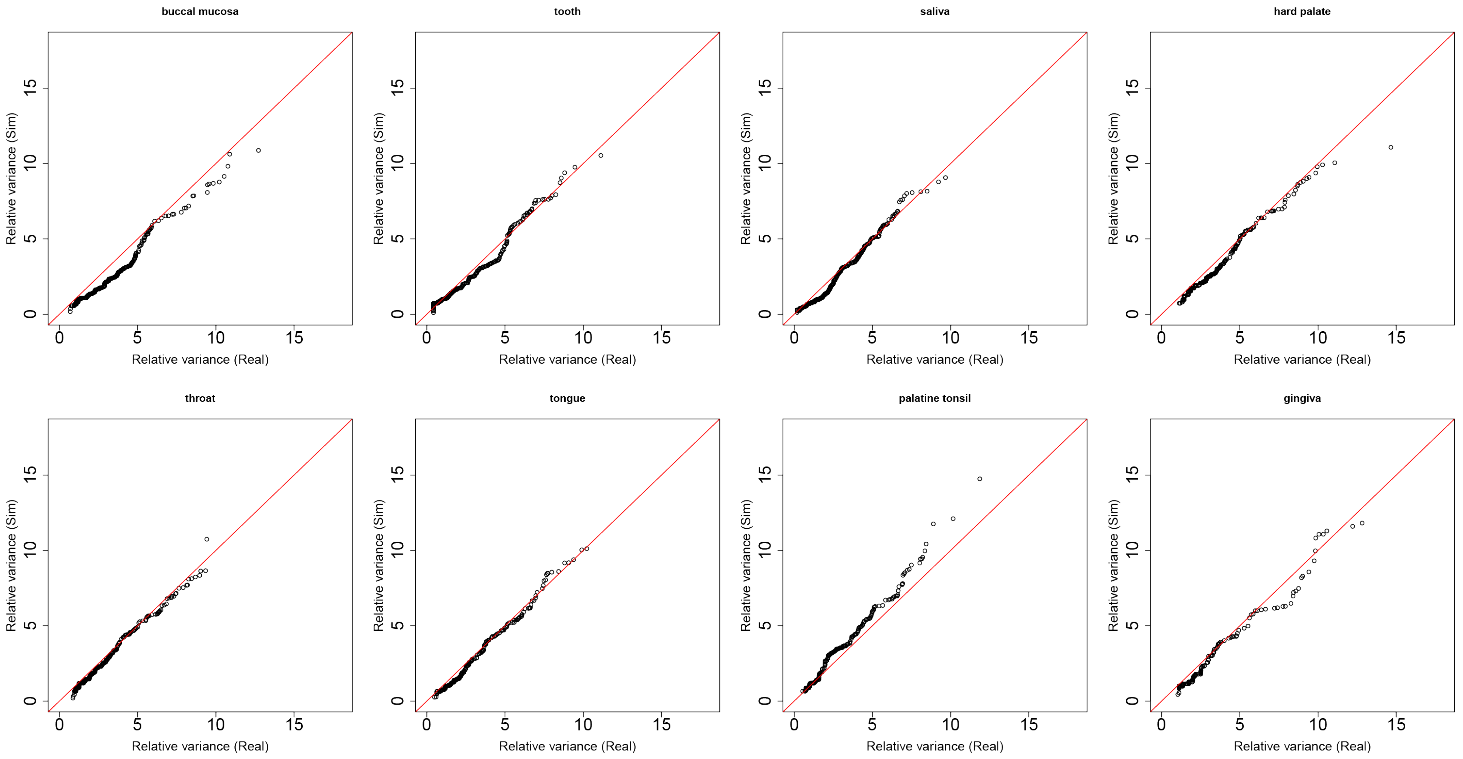

Figure 19 Q−Q plots of of Log2 RV values in real and simulated datasets within each group for HMP dataset, excluding zero mean features.

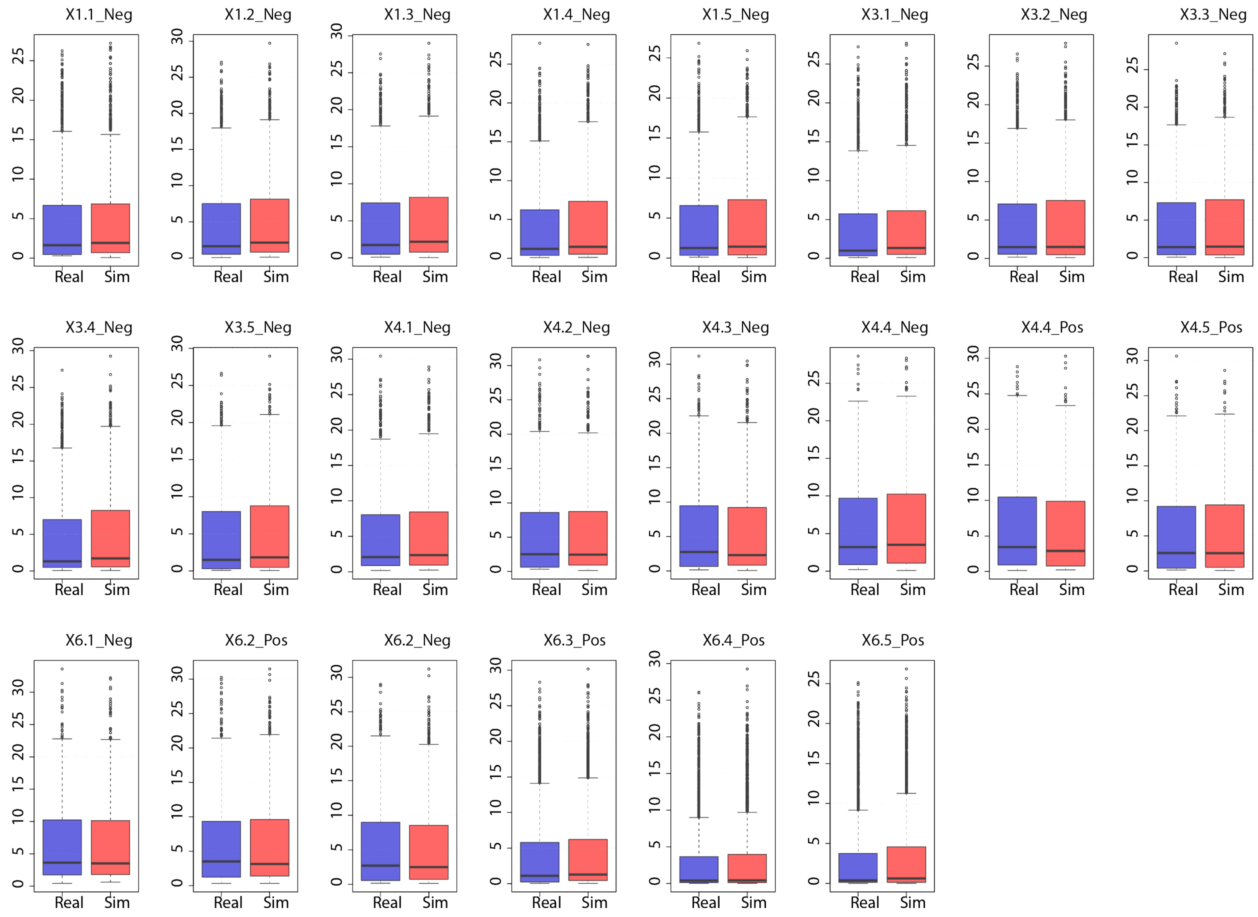

Figure 20 Box plots of of Log2 variance values in real and simulated datasets within each group for animal gut dataset, excluding zero mean features.

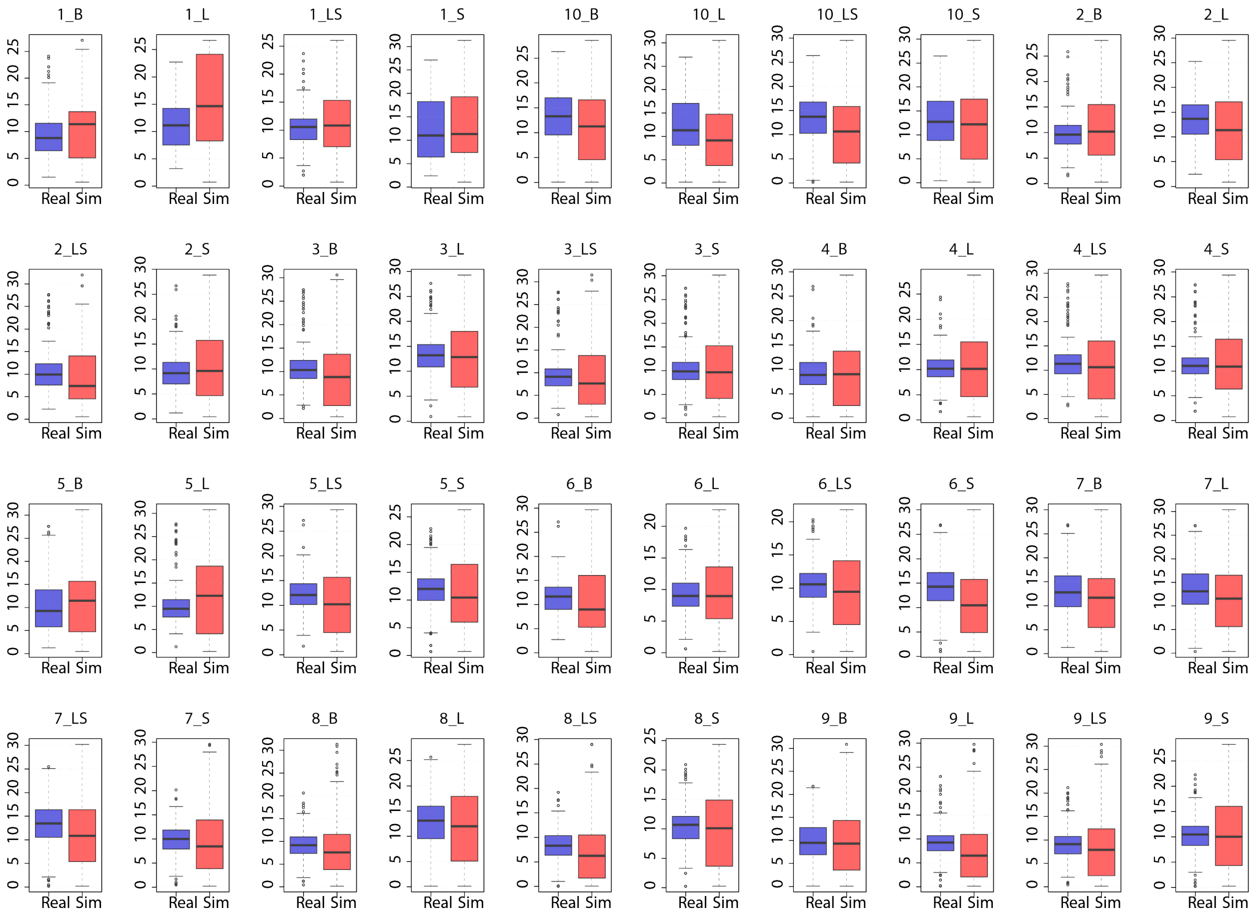

Figure 21 Box plots of of Log2 variance values in real and simulated datasets within each group for raw milk cheese dataset, excluding zero mean features.

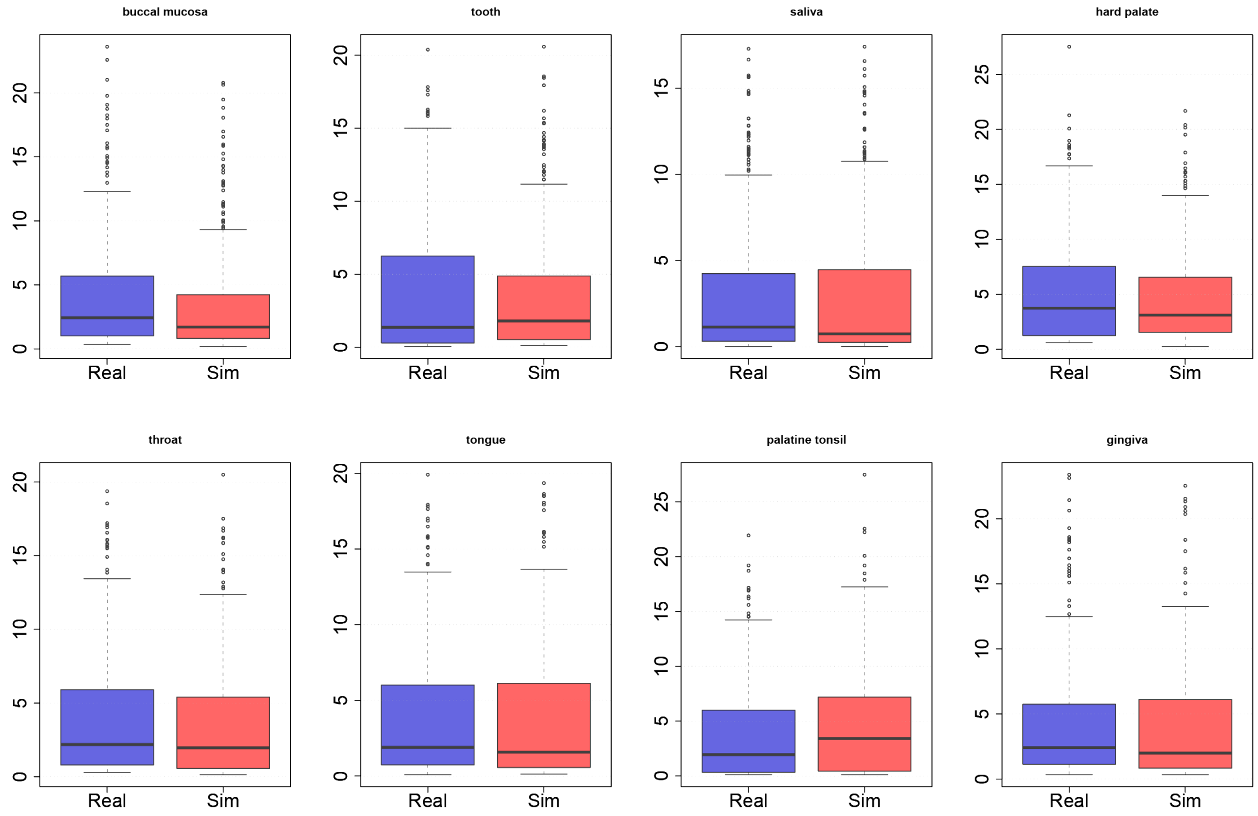

Figure 22 Box plots of of Log2 variance values in real and simulated datasets within each group for HMP dataset, excluding zero mean features.

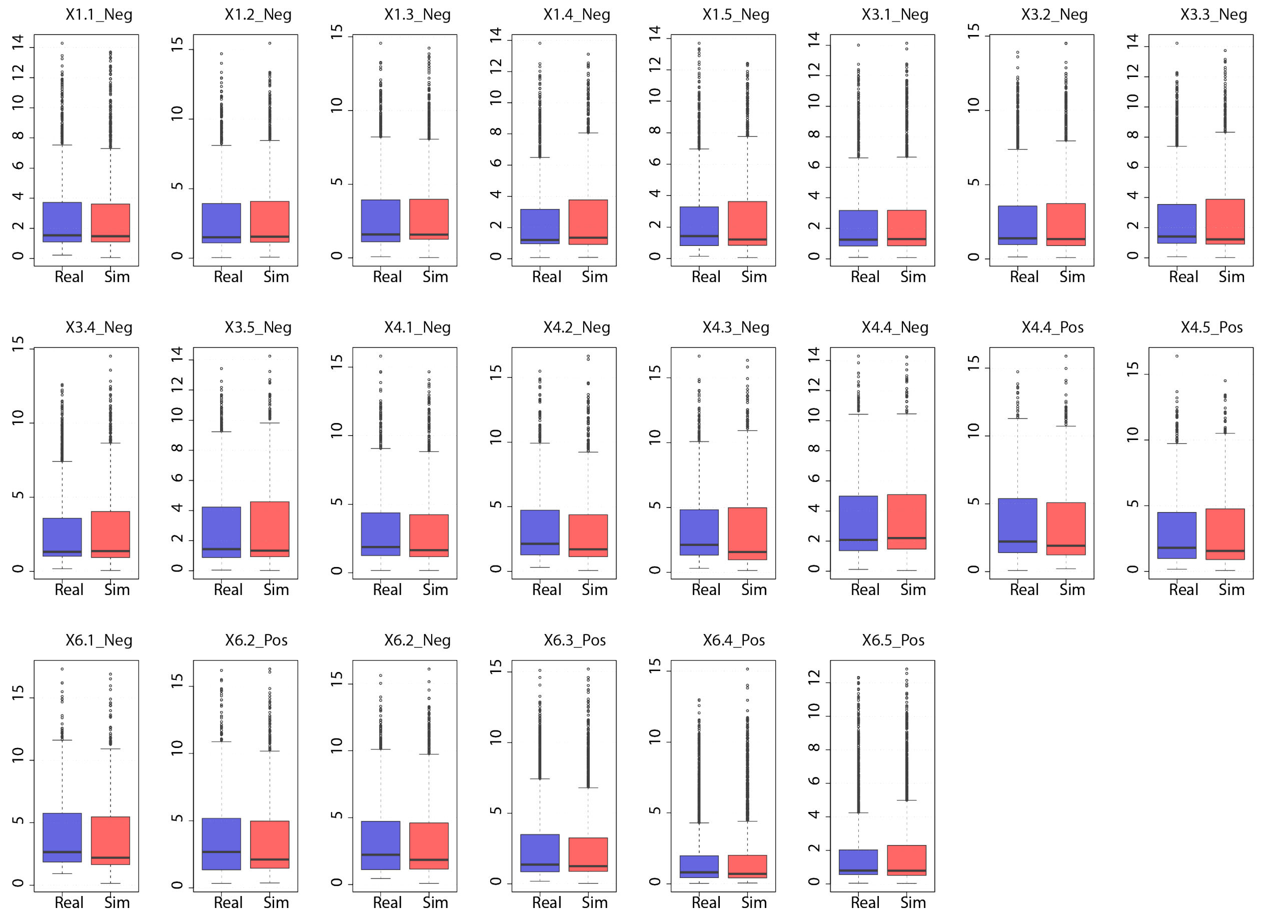

Figure 23 Box plots of of Log2 RV values in real and simulated datasets within each group for animal gut dataset, excluding zero mean features.

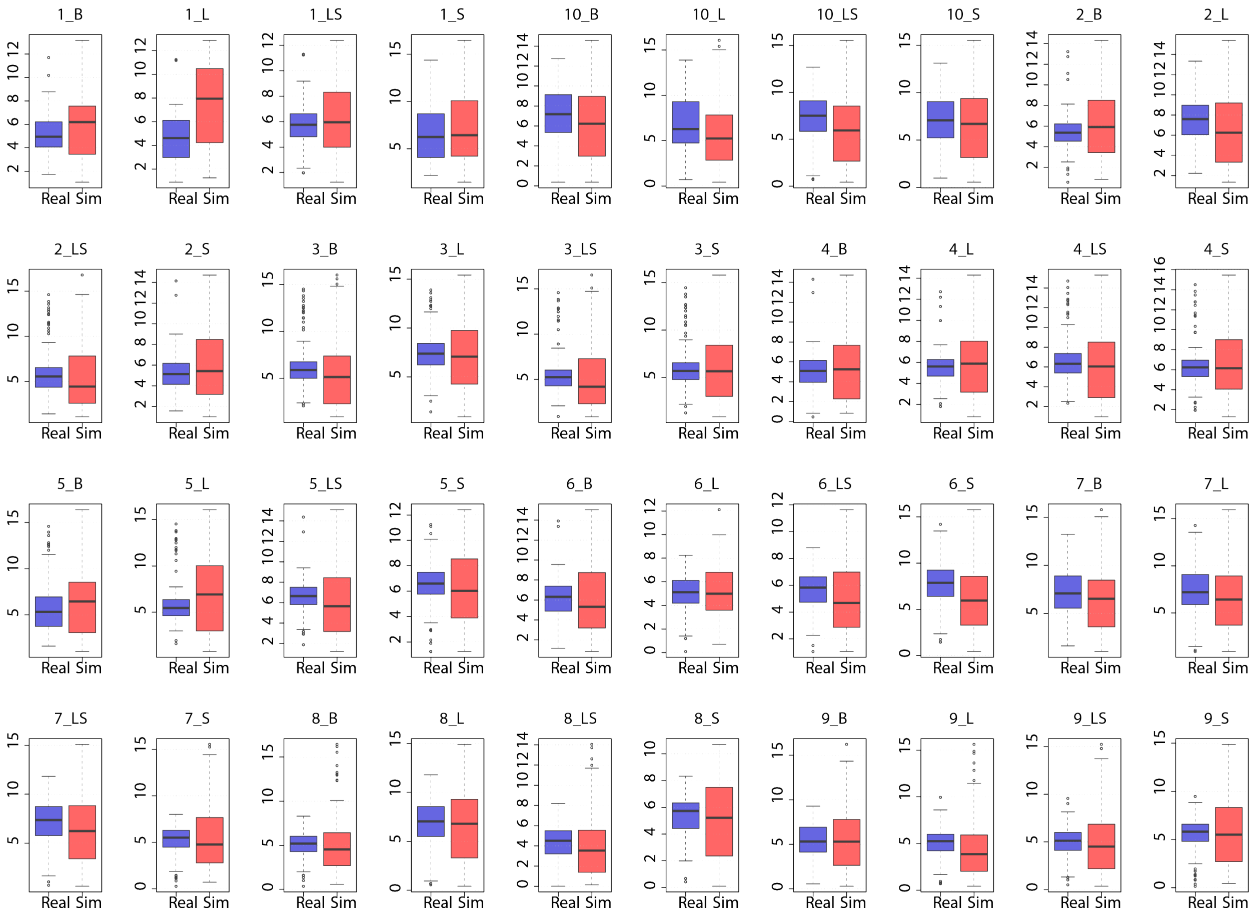

Figure 24 Box plots of of Log2 RV values in real and simulated datasets within each group for raw milk cheese dataset, excluding zero mean features.

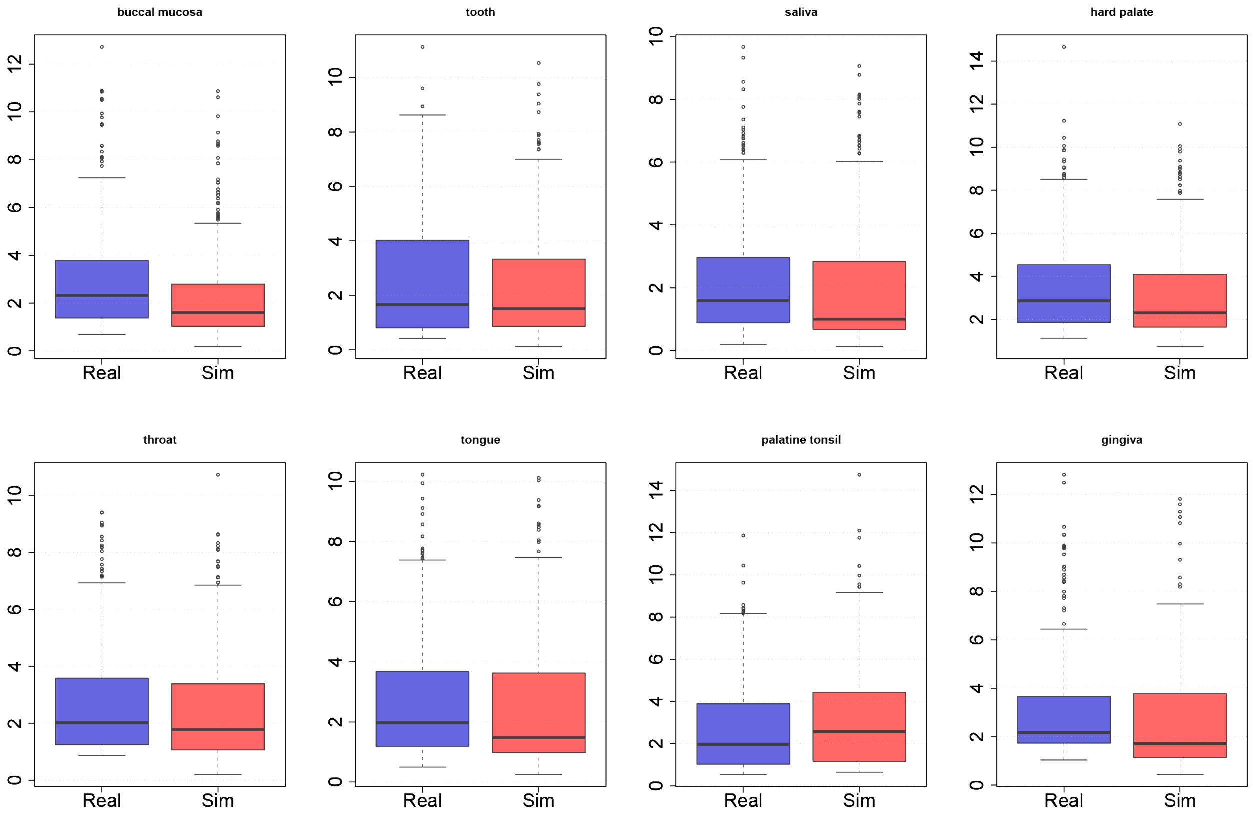

Figure 25 Box plots of of Log2 RV values in real and simulated datasets within each group for HMP dataset, excluding zero mean features.

Table 5 Mann-Whitney U tests and effect size results in comparing real and simulated variance distributions within groups for animal gut dataset.

Group	*P* value	Significance	Cohen's *d* magnitude	Bootstrap significance (%)
X1.1_Neg	0	Y	Negligible	1.71
X1.2_Neg	0	Y	Negligible	2.17
X1.3_Neg	0	Y	Negligible	7.82
X1.4_Neg	0	Y	Negligible	1.67
X1.5_Neg	0.11	N	—	0
X3.1_Neg	0	Y	Negligible	1.22
X3.2_Neg	0.517	N	—	0.01
X3.3_Neg	0.313	N	—	0
X3.4_Neg	0	Y	Negligible	1
X3.5_Neg	0	Y	Negligible	1.97
X4.1_Neg	0.03	Y	Negligible	0.14
X4.2_Neg	0.851	N	—	0
X4.3_Neg	0.403	N	—	0
X4.4_Neg	0.038	Y	Negligible	0.08
X4.4_Pos	0.083	N	—	0.03
X4.5_Pos	0.257	N	—	0
X6.1_Neg	0.241	N	—	0
X6.2_Pos	0.401	N	—	0.06
X6.2_Neg	0.274	N	—	0.08
X6.3_Pos	0	Y	Negligible	0.84
X6.4_Pos	0.041	Y	Negligible	0.03
X6.5_Pos	0	Y	Negligible	0.6

Table 6 Mann-Whitney U tests and effect size results in comparing real and simulated variance distribution within groups for raw milk cheese dataset.

Group	*P* value	Significance	Cohen's *d* magnitude	Bootstrap significance (%)
1_B	0.004	Y	Negligible	0.65
1_L	0.113	N	− − −	0.04
1_LS	0	Y	Negligible	5.86
1_S	0.008	Y	Negligible	0.47
10_B	0.001	Y	Negligible	0.17
10_L	0	Y	Negligible	2.13
10_LS	0	Y	Negligible	0.92
10_S	0	Y	Negligible	0.57
2_B	0	Y	Negligible	2.56
2_L	0	Y	Negligible	6.36
2_LS	0	Y	Negligible	4.12
2_S	0	Y	Negligible	1.25
3_B	0	Y	Negligible	8.87
3_L	0	Y	Negligible	3.06
3_LS	0	Y	Negligible	7.69
3_S	0	Y	Negligible	3.95
4_B	0	Y	Negligible	4.88
4_L	0.003	Y	Negligible	0.66
4_LS	0.002	Y	Negligible	0.34
4_S	0	Y	Negligible	3.54
5_B	0.005	Y	Negligible	0.75
5_L	0	Y	Negligible	2.17
5_LS	0	Y	Negligible	1.4
5_S	0	Y	Negligible	3.64
6_B	0	Y	Negligible	1.8
6_L	0	Y	Negligible	2.81
6_LS	0	Y	Negligible	2.26
6_S	0	Y	Negligible	2.07
7_B	0	Y	Negligible	7.29
7_L	0	Y	Negligible	3.45
7_LS	0.001	Y	Negligible	0.19
7_S	0	Y	Negligible	1.98
8_B	0	Y	Negligible	4.85
8_L	0	Y	Negligible	0.48
8_LS	0	Y	Negligible	0.68
8_S	0.002	Y	Negligible	0.44
9_B	0	Y	Negligible	0.48
9_L	0	Y	Negligible	4.09
9_LS	0.002	Y	Negligible	0.15
9_S	0	Y	Negligible	0.98

Table 7 Mann-Whitney U tests and effect size results in comparing real and simulated variance distribution within groups for HMP dataset.

Group	P value	Significance	Cohen's d magnitude	Bootstrap significance (%)
buccal mucosa	0	Y	Negligible	0.09
tooth	0.017	Y	Negligible	0.01
saliva	0.001	Y	Negligible	0.06
hard palate	0.007	Y	Negligible	0.02
throat	0.007	Y	Negligible	0
tongue	0.105	N	− − −	0
palatine tonsil	0	Y	Negligible	0.67
gingiva	0.08	N	− − −	0.01

References

[1] Robinson MD, McCarthy DJ, Smyth GK. edgeR: a Bioconductor package for differential expression analysis of digital gene expression data. Bioinformatics. 2010;26(1):139–140.

[2] Huttenhower C, Gevers D, Knight R, Abubucker S, Badger JH, Chinwalla AT, et al. Structure, function and diversity of the healthy human microbiome. Nature. 2012;486(7402):207.

[3] Methé BA, Nelson KE, Pop M, Creasy HH, Giglio MG, Huttenhower C, et al. A framework for human microbiome research. Nature. 2012;486(7402):215.

[4] Neilson JW, Califf K, Cardona C, Copeland A, van Treuren W, Josephson KL, et al. Significant Impacts of Increasing Aridity on the Arid Soil Microbiome. MSystems. 2017;2(3):e00195–16.

[5] Klindworth A, Pruesse E, Schweer T, Peplies J, Quast C, Horn M, et al. Evaluation of general 16S ribosomal RNA gene PCR primers for classical and next-generation sequencing-based diversity studies. Nucleic acids research. 2013;41(1):e1–e1.

[6] Andrews S. FastQC: a quality control tool for high throughput sequence data; 2010. Available from: `http://www.bioinformatics.babraham.ac.uk/projects/fastqc`.

[7] Caporaso JG, Kuczynski J, Stombaugh J, Bittinger K, Bushman FD, Costello EK, et al. QIIME allows analysis of high-throughput community sequencing data. Nature methods. 2010;7(5):335.

[8] DeSantis TZ, Hugenholtz P, Larsen N, Rojas M, Brodie EL, Keller K, et al. Greengenes, a chimera-checked 16S rRNA gene database and workbench compatible with ARB. Applied and Environmental Microbiology. 2006;72(7):5069–5072.

[9] Quast C, Pruesse E, Yilmaz P, Gerken J, Schweer T, Yarza P, et al. The SILVA ribosomal RNA gene database project: Improved data processing and web-based tools. Nucleic Acids Research. 2013;41(D1):590–596.

[10] Xie G, Lo CC, Scholz M, Chain PSG. Recruiting human microbiome shotgun data to site-specific reference genomes. PloS one. 2014;9(1):e84963.

[11] Wilk MB, Gnanadesikan R. Probability Plotting Methods for the Analysis of Data. Biometrika. 1968;55(1):1–17. Available from: http://www.jstor.org/stable/2334448.

[12] Tukey JW. Exploratory data analysis. vol. 2. Reading, Mass.; 1977.

[13] Phillips N. yarrr: A Companion to the e-Book "YaRrr!: The Pirate's Guide to R"; 2017. Available from: https://cran.r-project.org/package=yarrr.

[14] Mann HB, Whitney DR. On a Test of Whether one of Two Random Variables is Stochastically Larger than the Other. The Annals of Mathematical Statistics. 1947;18(1):50–60. Available from: http://projecteuclid.org/euclid.aoms/1177730491.

[15] Cohen J. Statistical Power Analysis for the Behavioral Sciences. Routledge; 1977.

[16] Sullivan GM, Feinn R. Using Effect Size - or Why the P Value Is Not Enough. Journal of Graduate Medical Education. 2012;4(3):279–282. Available from: http://www.jgme.org/doi/abs/10.4300/JGME-D-12-00156.1.

[17] Cano-Corres R, Sánchez-Álvarez J, Fuentes-Arderiu X. The Effect Size: Beyond Statistical Significance. Ejifcc. 2012;23(1):19–23.

[18] Coe R. It's the effect size, stupid. What effect size is and why it is important. British Educational Research Association annual conference. 2002;p. 1–18. Available from: http://www.cem.org/attachments/ebe/ESguide.pdf.

[19] Sawilowsky SS. New Effect Size Rules of Thumb. Journal of Modern Applied Statistical Methods. 2009;8(2):597–599. Available from: http://digitalcommons.wayne.edu/jmasm/vol8/iss2/26.
